# Cullin-3 and its adaptor protein ANKFY1 determine the surface level of integrin β1 in endothelial cells

**DOI:** 10.1242/bio.029579

**Published:** 2017-10-16

**Authors:** Masashi Maekawa, Kazufumi Tanigawa, Tomohisa Sakaue, Hiromi Hiyoshi, Eiji Kubota, Takashi Joh, Yuji Watanabe, Tomohiko Taguchi, Shigeki Higashiyama

**Affiliations:** 1 Division of Cell Growth and Tumor Regulation, Proteo-Science Center, Ehime University, Matsuyama 791-0295, Japan; 2 Department of Biochemistry and Molecular Genetics, Ehime University Graduate School of Medicine, Matsuyama 791-0295, Japan; 3 Department of Gastrointestinal Surgery and Surgical Oncology, Ehime University Graduate School of Medicine, Matsuyama 791-0295, Japan; 4 Department of Cardiovascular and Thoracic Surgery, Ehime University Graduate School of Medicine, Matsuyama 791-0295, Japan; 5 Department of Gastroenterology and Metabolism, Nagoya City University Graduate School of Medical Sciences, Nagoya City 467-8601, Japan; 6 Department of Health Chemistry, Graduate School of Pharmaceutical Science, University of Tokyo, Tokyo 113-0033, Japan; 7 Pathological Cell Biology Laboratory, Graduate School of Pharmaceutical Science, University of Tokyo, Tokyo 113-0033, Japan

**Keywords:** Cullin-3 (CUL3), Integrin, Membrane trafficking, Endothelial cells, Angiogenesis

## Abstract

Angiogenesis, the formation of new blood vessels from the pre-existing vasculature, is related to numerous pathophysiological events. We previously reported that a RING ubiquitin ligase complex scaffold protein, cullin-3 (CUL3), and one of its adaptor proteins, BAZF, regulated angiogenesis in the mouse retina by suppressing Notch signaling. However, the degree of inhibition of angiogenesis was made greater by CUL3 depletion than by BAZF depletion, suggesting other roles of CUL3 in angiogenesis besides the regulation of Notch signaling. In the present study, we found that CUL3 was critical for the cell surface level of integrin β1, an essential cell adhesion molecule for angiogenesis in HUVECs. By siRNA screening of 175 BTBPs, a family of adaptor proteins for CUL3, we found that ANKFY1/Rabankyrin-5, an early endosomal BTBP, was also critical for localization of surface integrin β1 and angiogenesis. CUL3 interacted with ANKFY1 and was required for the early endosomal localization of ANKFY1. These data suggest that CUL3/ANKFY1 regulates endosomal membrane traffic of integrin β1. Our results highlight the multiple roles of CUL3 in angiogenesis, which are mediated through distinct CUL3-adaptor proteins.

## Abstract (precis)

**Summary:** CUL3 and a CUL3-binding protein ANKFY1 determined the surface expression of integrin β1, an essential adhesion molecule for angiogenesis, by regulating the endosomal recycling traffic of integrin β1.

## INTRODUCTION

Angiogenesis (the process of new blood vessel growth) is not only essential to deliver oxygen and nutrients and dispose waste in the body for survival, but is also related to a variety of diseases (e.g. stroke, neurodegeneration, cancer, inflammatory disorders, hypertension, blinding eye diseases) (Carmeliet and Jain, 2011). Endothelial cells lining the inner surface of blood vessels change their characteristics by receiving various stimuli from soluble growth factors, extracellular matrix and neighboring cells directly, leading to angiogenesis (Potente et al., 2011). The molecular mechanisms of this complicated multistep process have been intensively elucidated (Carmeliet and Jain, 2011; Potente et al., 2011; Ramasamy et al., 2015). Among the multiple steps in angiogenesis, proliferative signals through vascular endothelial growth factor receptors (VEGFRs) and antiangiogenic signals through Notch receptors are strictly balanced as in a ‘tug-of-war’ (Eilken and Adams, 2010).

CUL3, one of the cullin family proteins, is complexed to a RING ubiquitin E3 ligase. The CUL3-RING ubiquitin E3 ligase recruits substrate proteins through BTBPs and mediates their ubiquitination (Petroski and Deshaies, 2005). CUL3 plays essential functions in various fundamental cellular events (e.g. cell cycle, membrane trafficking, transcription in stress and developmental signaling, cell death and cytoskeletal rearrangement), and the dysregulation of the CUL3-mediated ubiquitination system is related to human diseases such as metabolic diseases, cancer and dystrophy (Genschik et al., 2013). The human genome encodes 183 BTBPs, and this large repertoire of CUL3 adaptors may contribute to the diverse function of CUL3 (Stogios et al., 2005). We previously reported that mRNA expression of BAZF, one of the BTBPs, was upregulated by vascular endothelial growth factor (VEGF) stimulation in human umbilical vein endothelial cells (HUVECs) (Ohnuki et al., 2012). Knockdown of CUL3 or BAZF inhibited angiogenesis in HUVECs (Ohnuki et al., 2012). A CUL3/BAZF complex polyubiquitinated a nuclear protein CBF1 for its proteasomal degradation (Ohnuki et al., 2012), a protein that forms a complex with the intracellular domain of Notch (NICD) at the specific DNA sites to induce antiangiogenic genes that include HEY1 and HEY2 (Fischer et al., 2004). These results suggested that CUL3/BAZF function in the nucleus to regulate the transcription of certain genes for angiogenesis. However, the degree of inhibition of the angiogenesis was made more severe by CUL3 depletion than by BAZF depletion (Ohnuki et al., 2012; Sakaue et al. 2017b), suggesting other roles of CUL3 in angiogenesis besides the regulation of Notch signaling.

Integrins are a large family of cell surface receptors mediating cellular adhesion to the extracellular matrix proteins, such as collagen and fibronectin (Hynes, 2002b). Integrins exist as two noncovalently bound α and β subunits, which pair to form heterodimers. Humans have at least 18 α subtypes and eight β subtypes, which together generate 24 distinct integrin heterodimers (Hynes, 2002a). In addition to the VEGFR and Notch signaling that regulate angiogenesis, integrins in endothelial cells are essential for the proper progress of angiogenesis through the establishment of cell adhesion to the extracellular matrix (Carmeliet and Jain, 2011). Genetic deletion of integrin α5 or fibronectin, a ligand for integrin α5/β1, leads to embryonic lethality with severe vascular defects in mice (George et al., 1993; Yang et al., 1993), indicating that integrin α5/β1 and fibronectin are proangiogenic. Antagonists of integrin α5/β1 (e.g. antibodies and peptides) inhibited angiogenesis (Kim et al., 2000). Integrin α1/β1 and α2/β1, receptors for collagens, also function in angiogenesis. Both of these integrins are upregulated by VEGF, and antibodies to integrin α1/β1 and α2/β1 inhibit tumor angiogenesis (Senger et al., 1997). Localization of integrins on the plasma membrane (PM) is determined by the rate of their endocytosis and their recycling from endosomes to the PM (De Franceschi et al., 2015). As expected, Arf6 that regulates endosomal recycling of integrin β1 is essential for angiogenesis (Hongu et al., 2015).

In the present study, we show that CUL3 regulates the endosomal membrane traffic of integrin β1, and thus determines the cell surface level of integrin β1. We further show that ANKFY1 is a CUL3-associated BTBP, essential for the endosomal membrane traffic of integrin β1, cell spreading on the BM and angiogenesis *in vitro*.

## RESULTS

### CUL3 is required for localization of integrin β1 on the surface of endothelial cells

**Fig. 1.**
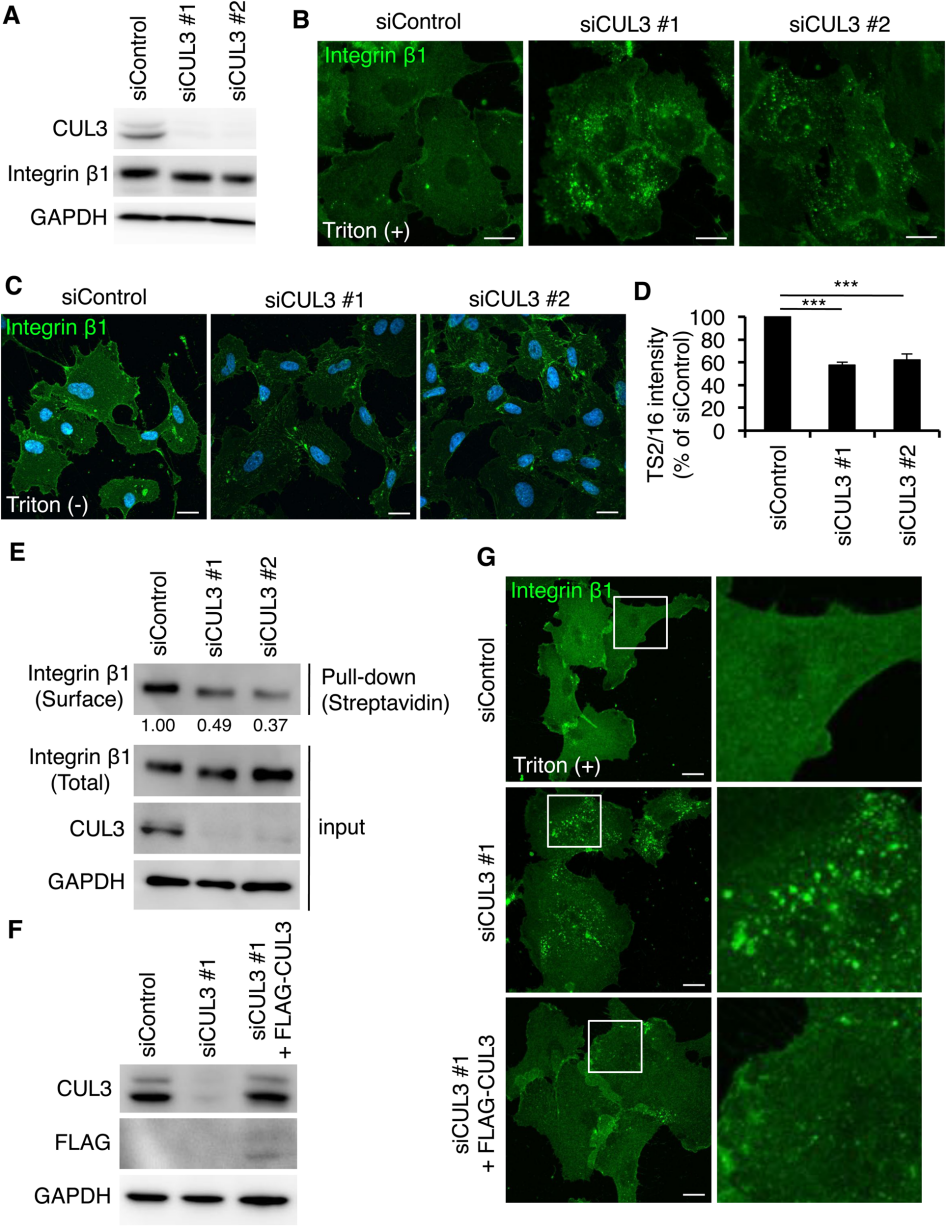
**CUL3 regulates localization of cell surface integrin β1 in HUVECs.** (A) Western blots of HUVEC lysates 72 h post-transfection of siRNAs. (B) Confocal images of HUVECs fixed after 72 h transfection of siRNA, permeabilized and stained for integrin β1 by P5D2. (C) Confocal images of HUVECs fixed after 72 h transfection of siRNA and stained for integrin β1 by Alexa488-conjugated TS2/16 without membrane permeabilization. (D) Quantitation of C; 50 cells from three independent experiments were analyzed. Data show the mean±s.e.m. ***P<0.001. (E) Surface proteins were biotinylated, collected with streptavidin beads, and the total and cell surface integrin β1 were detected by TS2/16. The numbers indicate band intensity of surface/total integrin β1 (normalized to siControl). (F,G) Rescue experiments of CUL3 knockdown. Western blots of HUVEC lysates (F) and confocal images of intracellular integrin β1 in HUVECs (G) infected with siRNA-resistant-FLAG-CUL3-carrying lentivirus. Integrin β1 was labelled with P5D2. Magnifications of the squared areas are shown on the right. Scale bars: 20 µm.

Given accumulating evidence that CUL3 functions in membrane trafficking (Gschweitl et al., 2016; Jin et al., 2012; Yuan et al., 2014), we examined whether CUL3 regulates the PM localization of integrin β1, an essential angiogenic factor in endothelial cells (Carlson et al., 2008; Hongu et al., 2015; Lei et al., 2008; Tanjore et al., 2008; Yamamoto et al., 2015; Zovein et al., 2010). As reported previously (Tiwari et al., 2011), in HUVECs, integrin β1 was mainly found on the PM (Fig. 1A,B). In contrast, in cells depleted of CUL3, integrin β1 was localized at intracellular punctate structures (Fig. 1A,B). The protein expression level of integrin β1 was not affected by CUL3 knockdown, suggesting that the localization of integrin β1 was shifted from the PM to intracellular compartments by CUL3 knockdown. We further confirmed the decrease in the cell surface integrin β1 levels on CUL3-knockdown cells by staining cells without membrane permeabilization (Fig. 1C,D), or by biotinylation of surface proteins (Fig. 1E). Both methods showed that the cell surface integrin β1 level was decreased to ∼40–50% by CUL3 knockdown. The expression of FLAG-tagged siRNA-resistant CUL3 diminished the intracellular punctate structures positive for integrin β1 in CUL3-knockdown cells (Fig. 1F,G), excluding the off-target effect of siRNA. These results indicated that CUL3 is essential for the cell surface integrin β1 localization in HUVECs.

### CUL3 regulates recycling of integrin β1 from endosomes to the plasma membrane

**Fig. 2.**
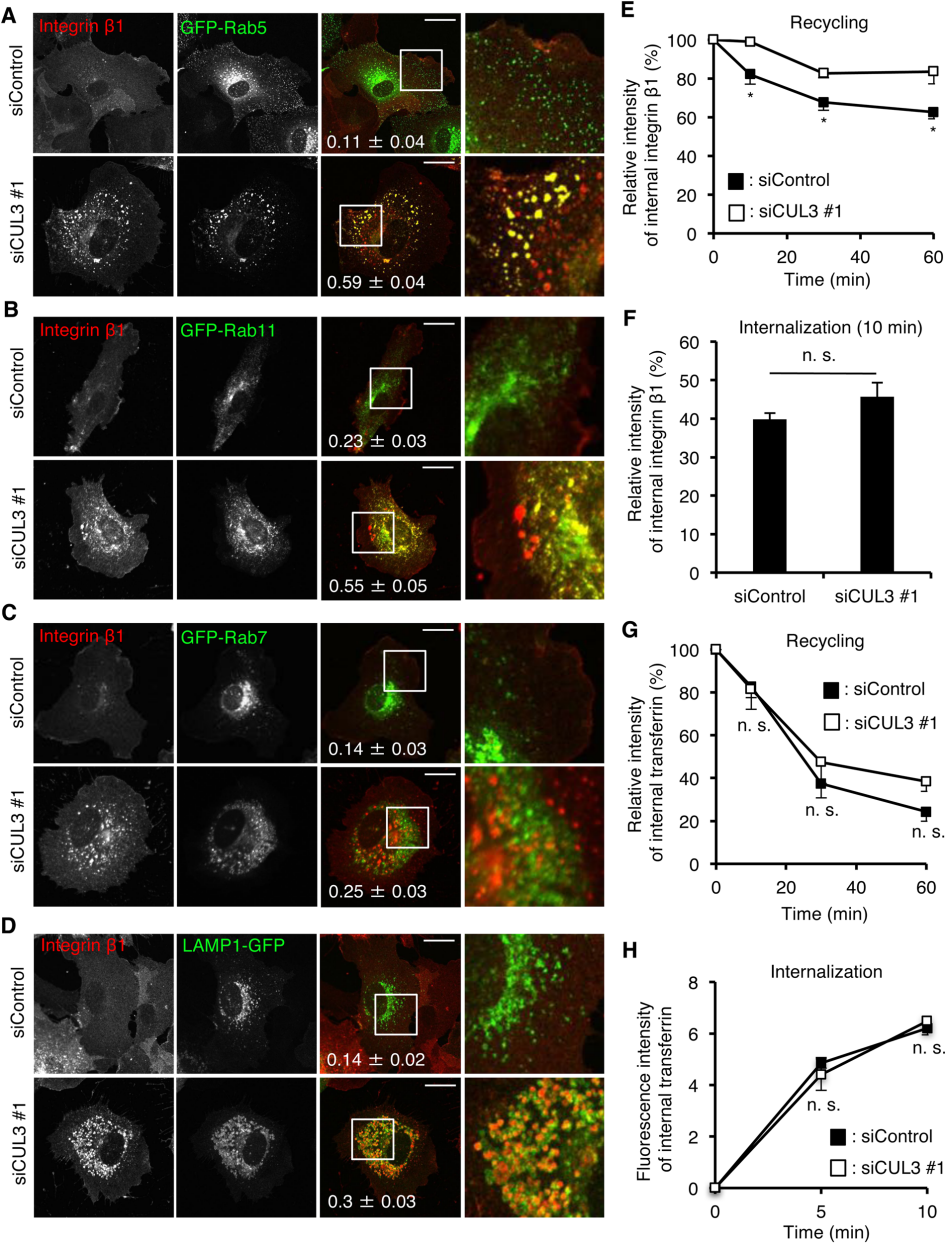
**CUL3 regulates the recycling pathway of integrin β1.** (A-D) Confocal images of control or CUL3 knockdown HUVECs expressing the GFP-tagged organelle markers Rab5 (A; for early endosomes), Rab11 (B; for recycling endosomes), Rab7 (C; for late endosomes) and LAMP1 (D; for lysosomes) by lentiviral infection. Magnifications of the squared areas are shown on the right. Pearson's correlation coefficient is indicated in the merged images. Thirty cells from three independent experiments were analyzed. Scale bars: 20 µm. (E) Recycling of integrin β1. HUVECs were treated with control siRNA and CUL3 siRNA #1 for 72 h. Representative images are shown in Fig. S1B. The fluorescence intensity of Alexa488-TS2/16 is shown as a percentage of that at 0 min. One hundred cells from three independent experiments were analyzed. Data show the mean±s.e.m. *P<0.05. (F) Internalization of integrin β1. HUVECs were treated with control siRNA and CUL3 siRNA #1 for 72 h. Surface integrin β1 was labeled with Alexa488-TS2/16 and chased in medium for 10 min. Representative images are shown in Fig. S1C. The fluorescence intensity of Alexa488-TS2/16 is shown as a percentage of that at 0 min (the fluorescence intensity on the cell surface before quenching). One hundred cells from three independent experiments were analyzed. Data show the mean±s.e.m. n. s., not significant. (G) Recycling of transferrin. HUVECs were treated with control siRNA and CUL3 siRNA #1 for 72 h. The fluorescence intensity of Alexa488-Tfn is shown as the percentage of that at 0 min. Fifty cells from three independent experiments were analyzed. Data show the mean±s.e.m. n. s., not significant. (H) Internalization of transferrin. HUVECs were treated with control siRNA and CUL3 siRNA #1 for 72 h. Cells were incubated with Alexa488-Tfn for 5 and 10 min, acid-washed and fixed. The fluorescence intensity of Alexa488-Tfn is shown. Fifty cells from three independent experiments were analyzed. Data show the mean±s.e.m. n. s., not significant.

We determined the intracellular compartments where integrin β1 was accumulated upon CUL3 knockdown. By comparing with the localizations of several endocytic proteins (Rab5, early endosomes; Rab11, recycling endosomes; Rab7, late endosomes; LAMP1, lysosomes), we found that integrin β1 localized mostly at early endosomes/recycling endosomes in CUL3-knockdown cells (Fig. 2A-D). We then compared the trafficking of integrin β1 in control and CUL3-knockdown cells. First, the recycling of integrin β1 from early endosomes/recycling endosomes to the PM was examined. Cells were labeled with Alexa488-conjugated anti-integrin β1 antibody (Alexa488-TS2/16) and incubated for 1 h. After quenching the surface integrin β1 with anti-Alexa488 antibody (time=0 in Fig. 2E; Fig. S1A,B), cells were further incubated for the indicated times, at which anti-Alexa488 antibody was added to the medium to quench the surface Alexa488/integrin β1, i.e., ‘recycled integrin β1’, and the total cellular intensity of Alexa488 was quantitated. As shown (Fig. 2E; Fig. S1A,B ), the intensity of Alexa488 in control cells after 60 min decreased to ∼70%, indicating that 30% of integrin β1 at endosomes at time=0 was recycled back to the PM. In contrast, the intensity of Alexa488 decreased to only 90% in cells depleted of CUL3 after 60 min. These results suggested that CUL3 knockdown significantly delayed the recycling of integrin β1 from endosomes to the PM. Second, the internalization of integrin β1 from the PM was examined. Cells were labeled with Alexa488-TS2/16 and chased for the indicated time to allow the labeled integrin β1 to be internalized. Anti-Alexa488 antibody was then added to the medium to quench the surface Alexa488/integrin β1. The remaining fluorescence of Alexa488 thus corresponded to integrin β1/Alexa488 that had been internalized during the chase period. As shown (Fig. 2F; Fig. S1A,C), the intensity of Alexa488 after the 10-min chase was ∼40% in both control and CUL3-knockdown cells, indicating that ∼40% of Alexa488/integrin β1 on the PM was internalized during the 10-min chase in these cells. The results of recycling and internalization assays suggested that CUL3 contributes specifically to the recycling pathway of integrin β1 from endosomes to the PM. The defect of recycling of integrin β1 from endosomes to the PM may account for the decrease in the level of integrin β1 at the PM in CUL3-knockdown cells.

We examined the trafficking of transferrin, which follows the endocytic recycling pathway as integrin β1. We observed no difference between control and CUL3-knockdown cells in either recycling or internalization of transferrin (Fig. 2G,H). These results suggested the cargo specificity of CUL3 in endocytic recycling.

### CUL3 regulates cell spreading on the basement membrane

**Fig. 3.**
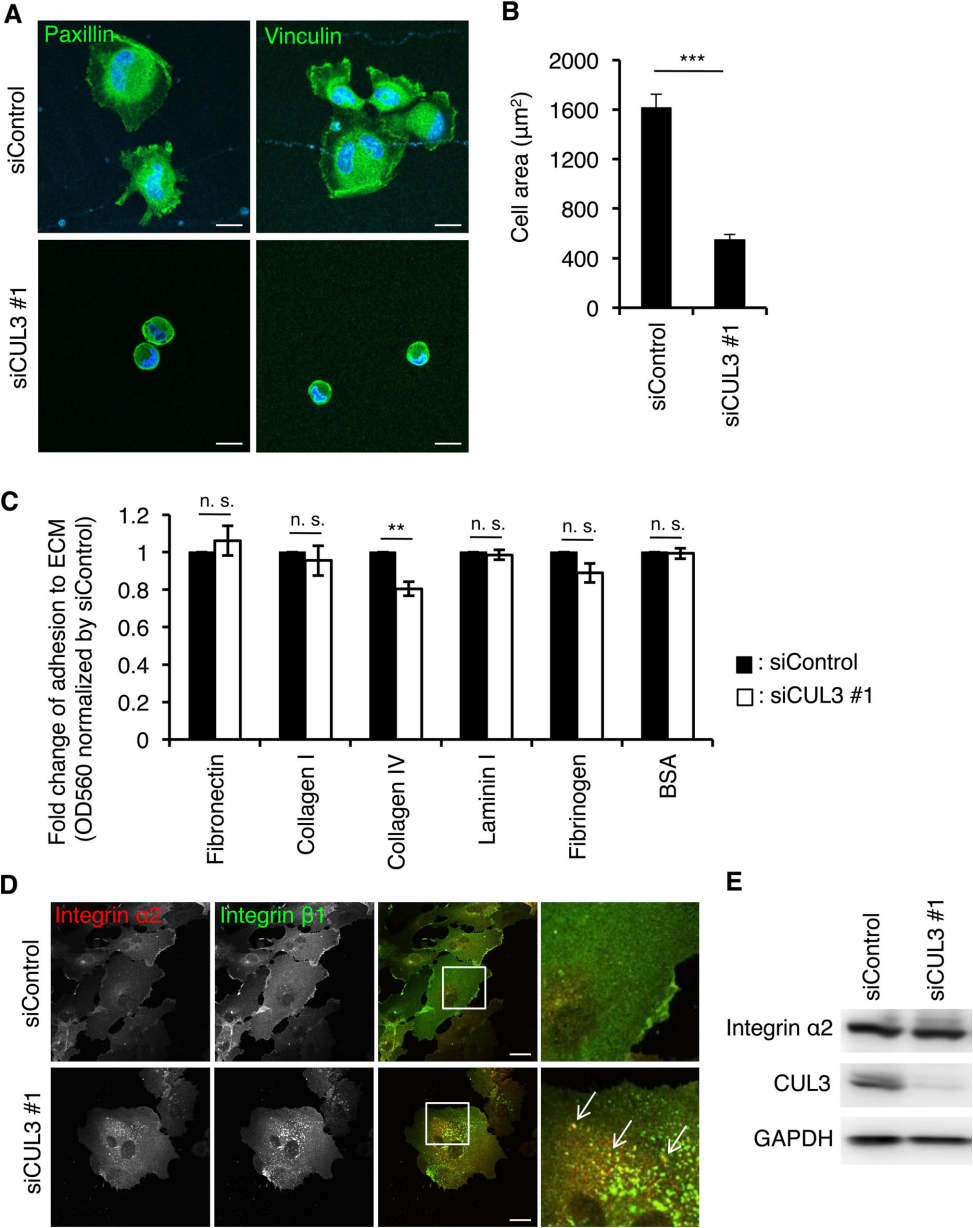
**Spreading of endothelial cells on the BM is impaired by knockdown of CUL3.** (A) Confocal images of HUVECs seeded on the BM and incubated for 1 h. Scale bars: 20 µm. (B) Areas of the cells in A; >70 cells from three independent experiments were analyzed. Data are mean±s.e.m. ***P<0.001. (C) Adhesion of HUVECs treated with control siRNA and CUL3 siRNA #1 to each extracellular matrix. Data were normalized to siControl and show the mean±s.e.m. of four independent experiments. **P<0.01, n. s., not significant. (D) Confocal images of intracellular integrin β1 and α2. HUVECs were fixed after 72 h transfection of siRNA. Magnifications of the squared areas are shown on the right. Representative colocalized integrin β1 and α2 are indicated by arrows. Scale bars: 20 µm. (E) Western blots of HUVEC lysates at 72 h post-transfection of siRNAs.

As CUL3 knockdown reduced the expression level of integrin β1 on the cell surface, a critical factor of cell adhesion to the extracellular matrix, we expected that CUL3-knockdown cells would be less adhesive to the extracellular matrix. To test this, we examined whether cell adhesion/spreading activity is affected by CUL3 knockdown, using BD Matrigel^™^ basement membrane (BM). BM contains extracellular matrix proteins including laminin, collagen IV, heparan sulfate proteoglycans and entactin/nidogen (Yurchenco, 2011). One hour after seeding on the BM, cells were fixed and stained for paxillin and vinculin, integrin-effector proteins that are essential for generating focal adhesions (Sun et al., 2016). As shown (Fig. 3A), control cells spread with typical lamellipodia, and paxillin and vinculin accumulated at the edge of the lamellipodia, indicating the formation of focal adhesions. In contrast, CUL3-knockdown cells remained mostly round without any lamellipodia. The spreading area of CUL3-knockdown cells decreased to 30% compared to that of control cells (Fig. 3B). These results suggested that CUL3 was required for the cell spreading activity on the BM. Using the CytoSelect™ 48-well Cell Adhesion Assay kit, in which individual wells are coated with pure extracellular matrix proteins, we found that CUL3-knockdown HUVECs were less adhesive to collagen IV (Fig. 3C). This result may be related to the fact that the BM contains collagen IV as a major component (Khoshnoodi et al., 2008; Yurchenco, 2011).

Integrin β1 can form heterodimers with 12 different integrin α subunits (Hynes, 2002a). Among the integrin α/β heterodimers, integrin α2/β1 binds to collagen IV (Keely et al., 1995; Kern et al., 1993). The decreased binding activity of cells to collagen IV by CUL3 knockdown suggested that integrin α2, like integrin β1, would be affected by CUL3 knockdown. We thus examined the subcellular localization of integrin α2. In control cells, integrin α2 was mainly found on the PM (Fig. 3D). In contrast, in CUL3-knockdown cells, integrin α2 localized at intracellular punctate structures, at which integrin β1 was partly colocalized (Fig. 3D, arrows). The protein expression level of integrin α2 was not affected by CUL3 knockdown (Fig. 3E), suggesting that the localization of integrin α2, like integrin β1, was shifted from the PM to intracellular compartments upon CUL3 knockdown. These results suggested that CUL3 is essential to determine the cell surface integrin α2/β1 heterodimer in HUVECs, thus contributing to cell adhesion to collagen IV.

### ANKFY1, a BTBP, is essential for the localization of integrin β1 on the cell surface and tube formation of HUVEC

**Fig. 4.**
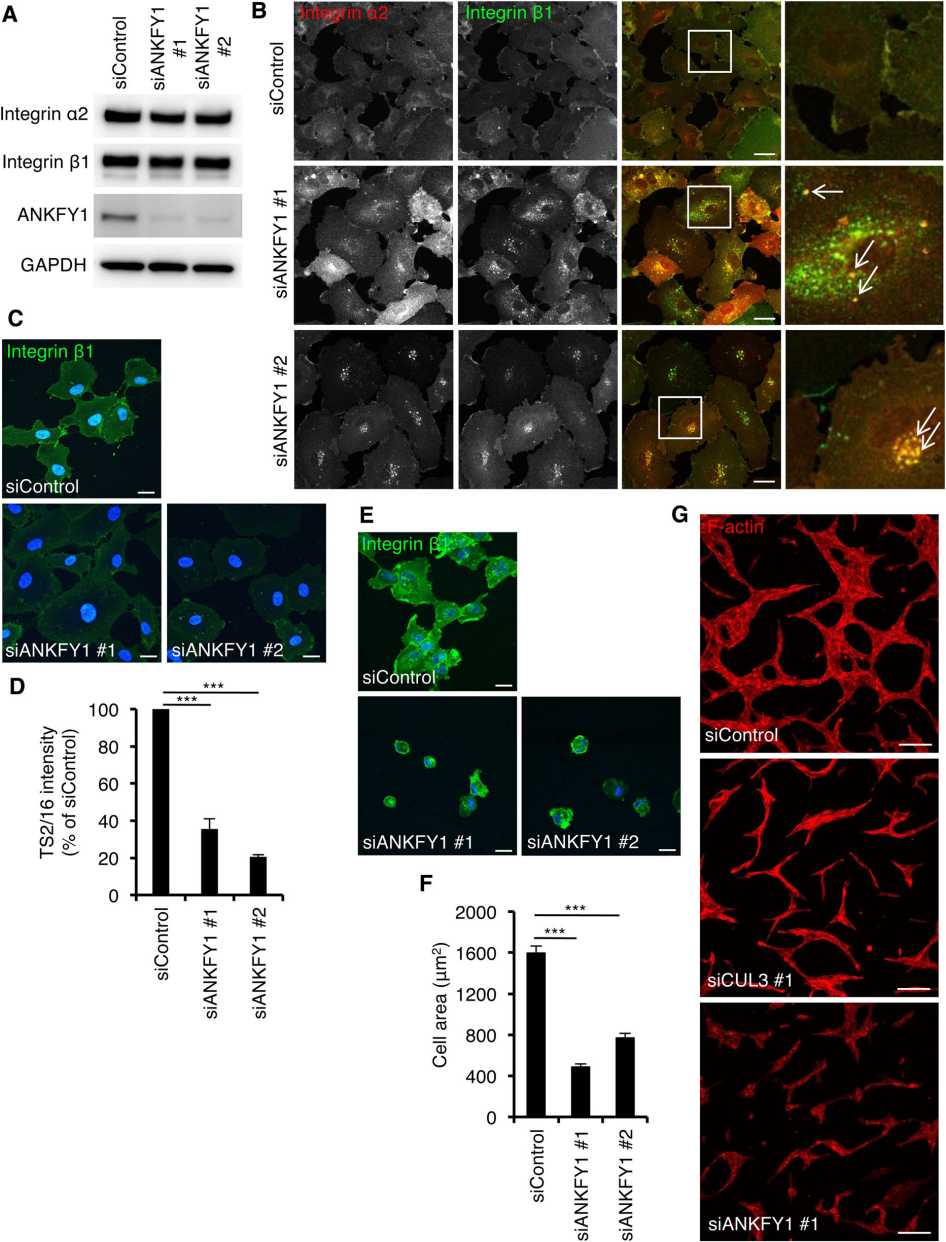
**ANKFY1 is a BTBP associating with CUL3 to regulate cellular distribution of integrin β1, cell spreading on the BM, and angiogenesis.** (A) Western blots of cell lysates of HUVECs at 72 h post-transfection of siRNAs. (B) Confocal images of intracellular integrin β1 and α2. HUVECs were fixed after 72 h transfection of siRNAs. Magnifications of the squared areas are shown on the right. Representative colocalized integrin β1 and α2 are indicated by arrows. (C) Confocal images of the cell surface integrin β1. HUVECs were fixed after 72 h transfection of siRNA and stained for integrin β1 by Alexa488-conjugated TS2/16 without membrane permeabilization. (D) Quantitation of C; 50 of cells from three independent experiments were analyzed. Data show the mean±s.e.m. ***P<0.001. (E) Confocal images of HUVECs spreading on the BM. HUVECs treated with control siRNA and ANKFY1 siRNA #1, #2 were seeded on the BM and incubated for 1 h. Bars; 20 µm. (F) Areas of the cells in E; >70 cells from three independent experiments were analyzed. Data show the mean±s.e.m. ***P<0.001. (G) Confocal images of tube formation. HUVECs seeded on collagen I gel were treated with control, CUL3 or ANKFY1 siRNA and packed on collagen I followed by VEGF stimulation for 66 h. Scale bars: 20 µm in B, C and E; 200 µm in G.

Recent studies suggest that members of the large family of BTBP (183 BTBPs in the human genome) define a new class of substrate-specific adaptors of CUL3 (Stogios et al., 2005). We sought to identify BTBP(s) that are essential for the cell surface localization of integrin β1. We performed siRNA screening against 175 BTBP genes (Table S1) and identified 7 BTBPs (ANKFY1, BTBD7, CCIN, KCTD2, KLHL18, KLHL34 and ZBTB6), the knockdown of which resulted in the intracellular accumulation of integrin β1, like that upon CUL3 knockdown (Fig. S2). Of note, the knockdown of BAZF, a BTBP that regulates angiogenesis by Notch-mediated transcriptional control in the nucleus (Ohnuki et al., 2012), did not affect the localization of integrin β1 (Fig. S2). Among these seven BTBPs, we particularly focused on ANKFY1, also known as Rabankyrin-5, because it localizes at early endosomes and regulates various endosomal membrane trafficking pathways (Nehru et al., 2013; Schnatwinkel et al., 2004; Zhang et al., 2012). ANKFY1 knockdown did not affect the protein expression level of integrin β1 and α2 (Fig. 4A). In ANKFY1-knockdown cells, as in CUL3-knockdown cells, integrin α2 localized at intracellular punctate structures, at which integrin β1 was partly colocalized (Fig. 4B, arrows). The expression of HA-tagged siRNA-resistant ANKFY1 diminished the intracellular punctate structures positive for integrin β1 in ANKFY1-knockdown cells (Fig. S3), excluding the off-target effect of siRNA. Staining of the surface integrin β1 indicated that the cell surface integrin β1 level decreased to ∼20–40% upon ANKFY1 knockdown (Fig. 4C,D). These results indicated that ANKFY1 is essential for the cell surface localization of integrin α2/β1 heterodimer in HUVECs. ANKFY1 knockdown drastically inhibited the cell adhesion to the BM (Fig. 4E), and the spreading area of ANKFY1-knockdown cells decreased to 30–50% compared to that of control cells (Fig. 4F). Critically, ANKFY1 knockdown, like CUL3 knockdown, inhibited the tube formations of HUVECs in an *in vitro* assay system that mimics angiogenesis *in vivo* (Arnaoutova and Kleinman, 2010) (Fig. 4G).

### Neddylation of CUL3 is essential for the interaction of CUL3 with ANKFY1

**Fig. 5.**
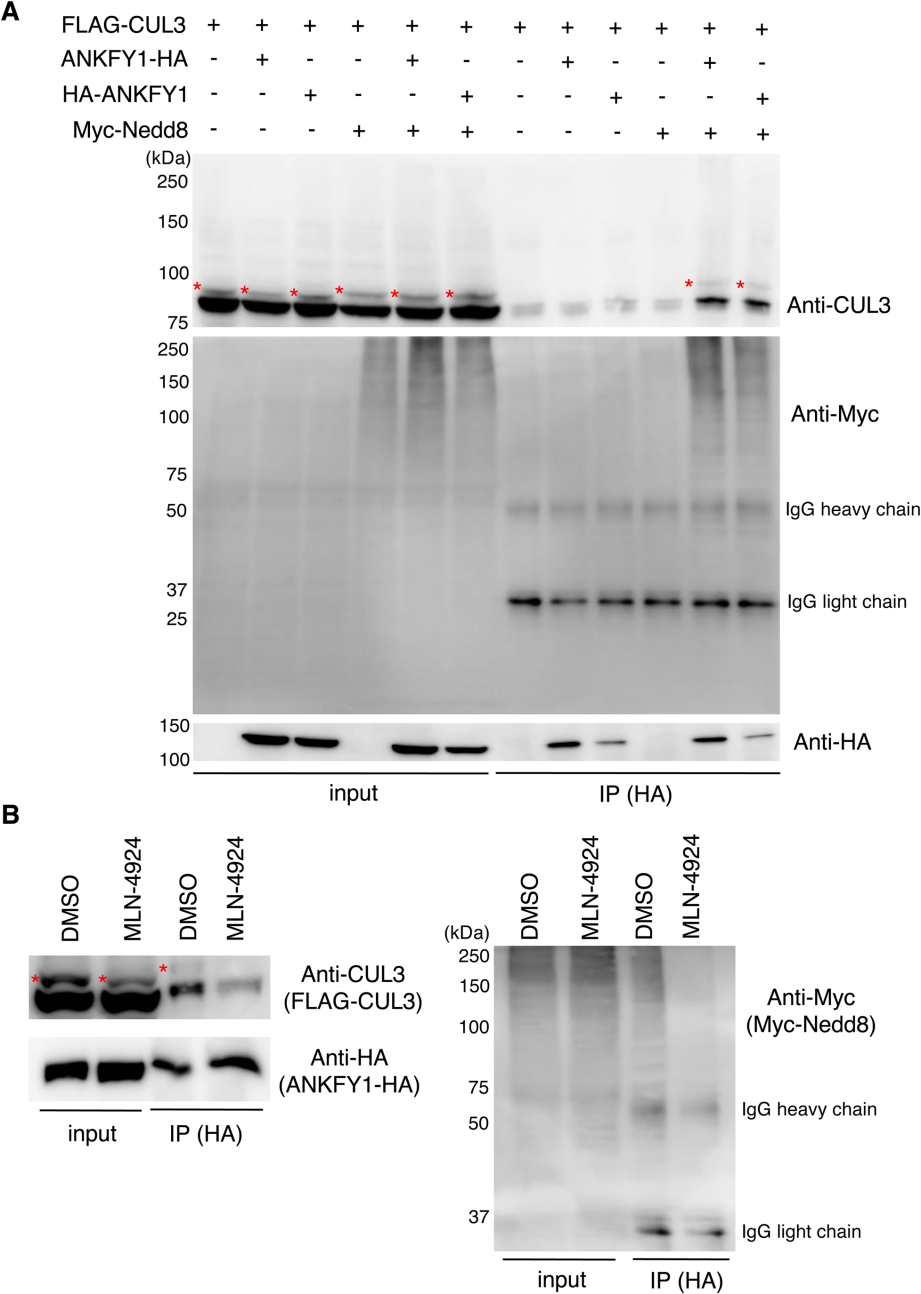
**Interaction of ANKFY1 and CUL3.** (A) FLAG-CUL3, ANKFY1-HA, HA-ANKFY1, Myc-Nedd8 and mock plasmid (pcDNA3.1) were expressed in HEK293T cells for 48 h. ANKFY1 tagged at its N terminus or C terminus with HA was expressed to validate the effects of the location of the tag on its interaction with CUL3. The lysates were then immunoprecipitated with anti-HA antibody. Total cell lysates (input) and immunoprecipitates (IP) were separated by SDS-PAGE and then blotted for CUL3 and HA. The asterisks indicate neddylated CUL3. IgG heavy and light chains are shown in the blot with anti-Myc antibody. (B) FLAG-CUL3, ANKFY1-HA and Myc-Nedd8 were expressed in HEK293T cells for 48 h. The lysates were then immunoprecipitated with anti-HA antibody. Total cell lysates (input) and IP were separated by SDS-PAGE, and then blotted for CUL3 and HA. Before cell lysis, HUVECs were treated with 1 µM MLN-4924 for 20 h. The asterisks indicate neddylated CUL3. IgG heavy and light chains are shown in the blot with anti-Myc antibody.

We then examined whether CUL3 interacted with ANKFY1. Given that neddylation, the process that conjugates the ubiquitin-like polypeptide Nedd8 to the conserved lysines of cullins, is essential for *in vivo* cullin-organized E3 activities (Wu et al., 2005), we expressed FLAG-tagged CUL3, HA-tagged ANKFY1, and Myc-tagged Nedd8 in HEK293T cells and examined the co-immunoprecipitation of CUL3 with HA-tagged ANKFY1. As shown (Fig. 5A), co-immunoprecipitation of CUL3 with ANKFY1 was detected when Myc-Nedd8 was co-expressed. In the immunoprecipitates, the neddylated CUL3 (indicated by asterisks) and non-neddylated CUL3 were present.

The significance of neddylation of CUL3 in the interaction with ANKFY1 was also suggested by the experiment using MLN-4924, a NAE1 (Nedd8-activating enzyme 1) inhibitor that reduces neddylation of cullin proteins, including CUL3 (Soucy et al., 2009). Treatment of HEK293T cells with MLN-4924 reduced the neddylation of CUL3 (Fig. 5B, input lanes) and the amount of CUL3 that was co-immunoprecipitated with ANKFY1 (Fig. 5B, IP lanes).

A previous study has shown that the treatment of HUVECs or mice with 1 µM MLN-4924 inhibited angiogenesis (Yao et al., 2014). After treatment of HUVECs with 1 µM MLN-4924 for 20 h, neddylated CUL3 disappeared (Fig. S4A, asterisk). The protein expression level of integrin β1 and α2 did not change with MLN-4924 treatment; however, their subcellular localizations were drastically shifted to intracellular punctate structures, at which they colocalized (Fig. S4B, arrows). MLN-4924 treatment inhibited the spreading of HUVECs on the BM (Fig. S4C,D). We then exploited the non-neddylated CUL3 mutant [CUL3(K712R)], in which the neddylation site of Lys712 is mutated to Arg (Wimuttisuk and Singer, 2007). The expression of siRNA-resistant CUL3 (K712R) could not restore the intracellular accumulation of integrin β1 in CUL3-knockdown cells (Fig. S4E,F). The results using CUL3 (K712R) and MLN-429 suggested that the neddylation of CUL3 is required for the cell surface localization of integrin β1 in HUVECs, and thus cell adhesion to the extracellular matrix.

### CUL3 is essential for endosomal localization of ANKFY1

**Fig. 6.**
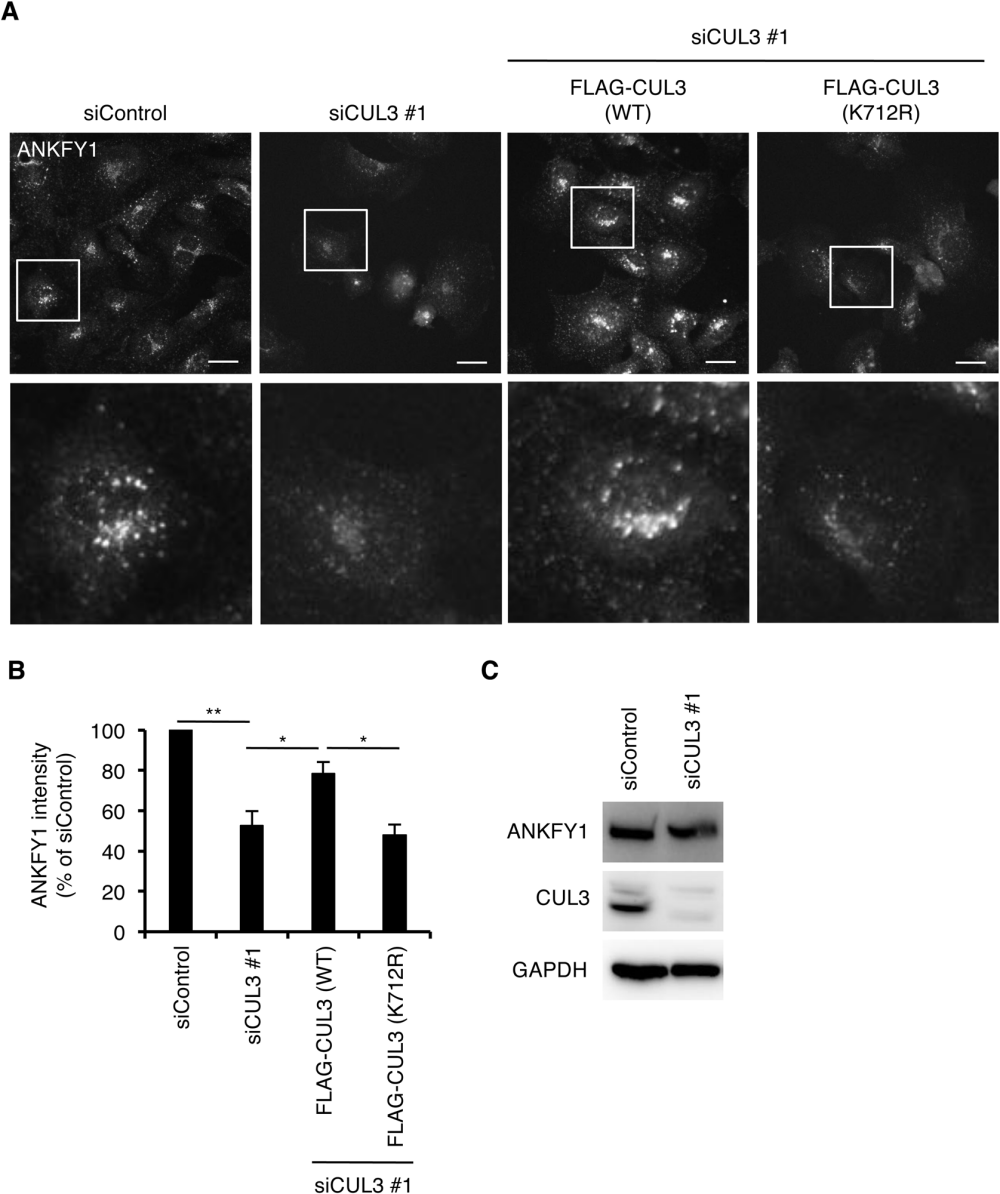
**CUL3 determines the endosomal localization of ANKFY1.** (A) Confocal images of HUVECs treated with control siRNA and CUL3 siRNA #1 for 72 h. CUL3-knockdown of HUVECs infected with siRNA of resistant-FLAG-CUL3 (WT or K712R)-carrying lentivirus for 48 h. Cells were fixed, permeabilized and stained for ANKFY1. Magnifications of the squared areas are shown in the lower panels. Scale bars: 20 µm. (B) Quantitation of A. The fluorescence intensities of ANKFY1 signals in 50 cells from three independent experiments were analyzed. Data show the mean±s.e.m. **P<0.01; *P<0.05. (C) Western blots of HUVEC lysates treated with control siRNA and CUL3 siRNA #1 for 72 h.

Finally, we examined whether the subcellular localization of ANKFY1 was regulated by CUL3. We compared the subcellular localization of endogenous ANKFY1 in control and CUL3-knockdown cells. In control HUVECs, ANKFY1 localized clearly at intracellular puncta structures (Fig. 6A), suggesting that ANKFY1 localized on early endosomal membranes as previously reported in A431 and NIH3T3 cells (Schnatwinkel et al., 2004). In contrast, in CUL3-knockdown cells, the membrane localization of ANKFY1 became less obvious. The fluorescence intensity of ANKFY1 in CUL3-depleted cells decreased by ∼50%, compared to that in the control cells (Fig. 6B). The protein level of ANKFY1 was not altered by CUL3 knockdown (Fig. 6C). These results suggested that a portion of ANKFY1 was relocated from endosomal membranes to the cytosol by CUL3 knockdown. The membrane localization of ANKFY1 was restored by the expression of siRNA-resistant CUL3 in CUL3-knockdown cells, but not by the expression of the siRNA-resistant non-neddylated CUL3 mutant [CUL3(K712R)] (Fig. 6A,B). These results suggested that CUL3 was critical for the endosomal membrane localization of ANKFY1 and that the neddylation of CUL3 was involved in this process.

## DISCUSSION

The role of CUL3 in membrane trafficking has recently been emerging, such as in the formation of COP-II vesicles (Jin et al., 2012) or anterograde transport carriers (Yuan et al., 2014) at the ER or trans-Golgi network, respectively. In particular, the study most relevant to the present findings is a report that CUL3 regulates the membrane trafficking of EGFR for degradation at endosomes (Huotari et al., 2012). Also, in HUVECs, knockdown of CUL3 drastically increased the cellular amount of EGFR (Fig. S5A), reinforcing the conserved role of CUL3 in the degradation of EGFR. Interestingly, however, the cellular amount of EGFR was not increased by knockdown of ANKFY1 (Fig. S5A). SPOPL is another BTBP that localizes at endosomes and negatively regulates the degradation of EGFR (Gschweitl et al., 2016). Unlike the knockdown of ANKFY1 (Fig. 4B), the knockdown of SPOPL did not result in the accumulation of integrin β1 at early/recycling endosomes (Fig. S5B). Thus, endosomes may harbor various CUL3-associated BTBPs so as to facilitate multiple endosomal trafficking pathways.

Knockdown of CUL3 resulted in the reduction of endosomal membrane localization of ANKFY1 without a change in its protein expression level (Fig. 6), suggesting that CUL3 is involved in the recruitment of ANKFY1 to early endosomes. By subfractionation experimentation, we noticed that the neddylated CUL3 (Fig. S6, asterisks) was exclusively recovered in the 100,000 ***g*** pellet fraction, but not in the 100,000 ***g*** supernatant fraction (cytosolic fraction). Given the critical contribution of the neddylation of CUL3 to the interaction of CUL3 and ANKFY1 (Fig. 5), neddylated CUL3, not non-neddylated CUL3, may function in recruiting ANKFY1 to early endosomes. ANKFY1 has a FYVE-finger domain at the *C*-terminus, which binds to phosphatidylinositol-3-phosphate (PI3P), a phosphoinositide abundant in early endosomal membranes (Schnatwinkel et al., 2004). ANKFY1 also binds to the GTP form (active form) of small GTPase Rab5, a crucial regulator of early endosome functions (Schnatwinkel et al., 2004). Whether CUL3 regulates the amount of PI3P and the active form of Rab5 remains to be elucidated.

Internalized integrins are trafficked to early endosomes, where cargoes are sorted for degradation or recycling (De Franceschi et al., 2015). The majority of internalized integrins are recycled back to the PM by the Rab4-dependent ‘short-loop’ pathway and/or the Rab11-dependent ‘long-loop’ recycling pathway through recycling endosomes (De Franceschi et al., 2015). In CUL3-depleted cells, we found the significant colocalization of integrin β1 with Rab5 (an early endosomal protein) and Rab11 (a recycling endosomal protein) (Fig. 2A,B). These results suggested that integrin β1 follows both short-loop and long-loop pathways in HUVECs and that CUL3 functions in early endosomes and recycling endosomes. CUL3 knockdown did not largely affect the internalization or recycling of transferrin receptor (TfnR) (Fig. 2G,H). Although there are shared molecular mechanisms between the recycling of integrin and TfnR, there are also integrin-specific regulators that provide spatial and temporal control of adhesion receptor availability at the PM: for example, in carcinoma cells, Rab-coupling protein, a member of the Rab11-family interacting proteins, has a crucial role in regulating integrin recycling but is dispensable for TfnR recycling (Muller et al., 2009). Such a protein, which specifically regulates the recycling of integrin β1, may require ubiquitination for its function and a CUL3/ANKFY complex would be responsible for this process.

Angiogenesis plays a critical role in the growth and metastasis of cancer. Blood supply is necessary for tumors to grow beyond a few millimeters in size. Antiangiogenic drugs such as Bevacizumab and Sunitinib that target VEGF and VEGFRs, respectively, have been successfully used to cure cancers; however, development of drug resistance after long-term administration is a common problem (Bergers and Hanahan, 2008; Lupo et al., 2016). CUL3 is essential for angiogenesis, and has been one of the promising targets for anticancer agents (Ohnuki et al., 2012; Yao et al., 2014; Zhao and Sun, 2013). However, the molecular mechanism by which CUL3 regulates angiogenesis is poorly understood. In the present study, we found that CUL3 and a CUL3-binding protein ANKFY1 determined the surface expression of integrin β1, an essential adhesion molecule for angiogenesis, by regulating the endosomal recycling traffic of integrin β1. Our results may provide a way to develop new antiangiogenic agents that specifically suppress some of the CUL3-downstream pathways involved in angiogenesis.

## MATERIALS AND METHODS

### Antibodies

The following antibodies were purchased from the manufacturers as indicated: goat anti-integrin β1 antibody (N-20, dilution 1:1000 for western blotting; Santa Cruz Biotechnology), mouse anti-integrin β1 antibody (P5D2, dilution 1:1000; R&D Systems), mouse anti-integrin β1 antibody (TS2/16, dilution 1:500 for western blotting and immunofluorescence; BioLegend, San Diego, USA), mouse anti-CUL3 antibody (CUL3-9, dilution 1:1000; Sigma-Aldrich), Alexa488-conjugated mouse anti-integrin β1 antibody (TS2/16, dilution 1:200; BioLegend), rabbit anti-integrin α2 antibody (EPR17338, dilution 1:6000 for western blotting, dilution 1:1000 for immunofluorescence; Abcam), mouse anti-ANKFY1 antibody (B-6, dilution 1:100 for western blotting, dilution 1:50 for immunofluorescence; Santa Cruz Biotechnology), mouse anti-paxillin antibody (349, dilution 1:200; BD Bioscience), mouse anti-vinculin antibody (hVIN-1, dilution 1:1000; Sigma-Aldrich), rabbit anti-EGFR antibody (D38B1, dilution 1:1000; Cell Signaling Technology), mouse anti-Calnexin antibody (ab2798, dilution 1:1000; Abcam), mouse anti-GAPDH antibody (5A12, dilution 1:6000; Wako, Tokyo, Japan), rabbit anti-HA antibody (Y-11, dilution 1:1000 for western blotting and immunofluorescence, dilution 1:100 for immunoprecipitation; Santa Cruz Biotechnology), mouse anti-Myc antibody (9E10, dilution 1:1000; Santa Cruz Biotechnology), mouse anti-FLAG antibody (M2, dilution 1:1000; Sigma-Aldrich), rabbit anti-Alexa488 antibody (A-11094, dilution 1:200; Thermo Fisher Scientific), HRP-conjugated anti-mouse IgG antibody (W4021, dilution 1:2000; Promega, Madison, USA), HRP-conjugated anti-rabbit IgG antibody (W4011, dilution 1:2000; Promega), HRP-conjugated anti-goat IgG antibody (V805A, dilution 1:2000; Promega), goat Alexa488-conjugated anti-mouse IgG antibody (A11001, dilution 1:2000; Molecular Probes, Eugene, USA), donkey Alexa488-conjugated anti-rat IgG antibody (A21208, dilution 1:2000; Molecular Probes), goat Alexa568-conjugated anti-mouse IgG antibody (A11004, dilution 1:2000; Molecular Probes), and goat Cy3-conjugated anti-rabbit IgG antibody (111-165-144, dilution 1:2000; Jackson ImmunoResearch Laboratories).

### Plasmids

GFP-Rab5, GFP-Rab7, GFP-Rab11, and LAMP1-GFP were amplified with vectors kindly provided by Dr. Gregory D Fairn (St. Michael's Hospital, Toronto, Canada) using the following pairs of primers: 5′-ATGGTGAGCAAGGGCGAGGA-3′ (GFP sense primer), 5′-TTAGTTACTACAACACTGATT-3′ (Rab5 antisense primer), 5′-TCAGCAACTGCAGCTTTCTGC-3′ (Rab7 antisense primer), 5′-TTAGATGTTCTGACAGCACTG-3′ (Rab11 antisense primer), 5′-ATGGCGGCCCCCGGCAGCGCC-3′ (LAMP1 sense primer), and 5′-TTACTTGTACAGCTCGTCCATGCCG-3′ (GFP antisense primer). The PCR products were introduced into the blunt end of the CSII-CMV-MCS-IRES2-Bsd vector. FLAG-CUL3 in the CSII-CMV-MCS-IRES2-Bsd vector (Sakaue et al., 2017a) was used for the rescue experiments. FLAG-CUL3 in the pcDNA3.1 vector was a kind gift from Dr. Takeshi Imamura (Ehime University, Matsuyama, Japan). FLAG-CUL3 K712R was generated with the following pairs of primers: 5′-GGATAATGAGATCTAGAAAGAAGAT-3′ (sense primer) and 5′-GCACTATAGCAGCTTCTATCTCATG-3′ (antisense primer). ANKFY1-HA and HA-ANKFY1 were amplified with cDNA derived from the human MGC clone using the following pairs of primers: 5′-ATGCCGACCCCGCGGGACTG-3′ (ANKFY1-HA sense primer) and 5′-CTAAGCGTAATCTGGAACATCGTATGGGTAAGAAACCCCACCCAGAGTCA-3′ (ANKFY1-HA antisense primer), 5′-ATGTACCCATACGATGTTCCAGATTACGCTATGCCGACCCCGCGGGACTG-3′ (HA-ANKFY1 sense primer), and 5′-CTAAGAAACCCCACCCAGAG-3′ (ANKFY1 antisense primer). The PCR products were introduced into the blunt end of the CSII-CMV-MCS-IRES2-Bsd vector or pcDNA3.1 vector. Myc-mouse Nedd8 in the pcDNA3.1 vector was a kind gift from Dr Kazuhiro Iwai (Kyoto University, Kyoto, Japan).

### Cell culture

Human umbilical vein endothelial cells (HUVECs) were purchased from Cell Systems (Kirkland, USA) and Lonza (Basel, Switzerland). HUVECs were maintained at 37°C with 5% CO_2_ in EBM-2 (Lonza) according to the manufacturer's instructions. HUVECs at passage 2–4 were used for experiments. HEK293T cells were maintained at 37°C with 5% CO_2_ in DMEM (Wako) supplemented with 10% fetal bovine serum (FBS), 20 units/ml penicillin and 100 µg/ml streptomycin. To inhibit the neddylation, HUVECs and HEK293T cells were treated with 1 µM MLN-4924 (Boston Biochem, Cambridge, MA, USA) at 37°C for 20 h.

### Transfection

For transfections of plasmids into HEK293T cells, GeneJuice (Millipore) was used according to the manufacturer's instructions. At 48 h post-transfection, cells were subjected to the subsequent experiments. Transfections of siRNAs (5–25 nM) into HUVECs were performed using RNAimax (Invitrogen) according to the manufacturer's instructions. Subsequent experiments were performed at 72 h post-transfection.

### siRNAs

The following validated siRNA duplex oligomers were purchased and used for knockdown experiments: GAGUGUAUGAGUUCCUAUU (siCUL3 #1, Sigma-Aldrich), GAAUAACAGUGGUCUUAGU (siCUL3 #2, Sigma), GCUGGAGCUUGUUACACAAAGGAAU (siANKFY1 #1, Invitrogen), GUACAGCGAUCUGAAGAUA (siANKFY1 #2, Thermo), GCAUUGUCGGUGAUUCCAA (siANKFY1 #3, targeting 3′-UTR of human ANKFY1 mRNA, Sigma-Aldrich). Control siRNAs were purchased from Sigma-Aldrich (SIC-001) or Invitrogen (12935-300).

### BTBP siRNA screening

The validated siRNA duplex oligomers array targeting 175 genes of BTBP was purchased from Thermo Fisher Scientific. HUVECs were seeded on cover slips in each well of 12-well plates (2×10^4^ cells/well). The next day, transfections of siRNAs (5 nM) into HUVECs were carried out using RNAimax (Invitrogen) according to the manufacturer's instructions. At 72 h post-transfection, cells were fixed with 4% paraformaldehyde (PFA) in PBS followed by immunofluorescence staining to visualize integrin β1 labelled with anti-integrin β1 antibody (P5D2; R&D Systems). The lists of BTBP genes and positive genes of which the knockdown showed relocation of integrin β1 in cellular compartments are shown in Table S1.

### Lentiviral expression

Lentiviruses carrying GFP-Rab5, GFP-Rab7, GFP-Rab11, LAMP1-GFP, FLAG-CUL3 (WT), FLAG-CUL3 (K712R) or ANKFY1-HA were produced by transfection of those cDNA cloned into the CSII-CMV-MCS-IRES2-Bsd vector with two packaging vectors (the pCAG-HIVgp vector and pCMV-VSVG-RSV-Rev vector) in HEK293T cells. At 48 h post-transfection, lentiviruses in medium were collected. At 24 h post-transfection of siRNA in HUVECs, the collected lentiviruses were added into the medium of the HUVECs. Expression of GFP-tagged organelle markers, FLAG-CUL3 (WT), FLAG-CUL3 (K712R), and ANKFY1-HA in HUVECs was detected at 48 h post-lentiviral infection. The CSII-CMV-MCS-IRES2-Bsd, pCAG-HIVgp, and pCMV-VSVG-RSV-Rev vectors were kind gifts from Dr. Hiroyuki Miyoshi (RIKEN, Tsukuba, Japan).

### Western blotting

Proteins were subjected to sodium dodecyl sulfate-poly acrylamide gel electrophoresis (SDS-PAGE) and transferred to polyvinylidene difluoride membranes (BioRad). After blocking in 5% skim milk in 0.05% Tween-20/TBS buffer, the membranes were incubated with primary antibodies, followed by secondary antibodies conjugated to peroxidase in 5% skim milk in 0.05% Tween-20/TBS buffer. The proteins were visualized by enhanced chemiluminescence using a LAS-4000 (GE Healthcare). To detect integrin β1 using TS2/16, cell lysates were collected in the nonreducing conditions. In experiments using TS2/16, all cell lysates were collected in the reducing conditions.

### Immunoprecipitation (IP)

HEK293T cells cultured in six-well plates were transfected with Myc-mouse Nedd8, FLAG-CUL3 and ANKFY1-HA (or HA-ANKFY1). At 48 h post-transfection, HEK293T cells were washed with ice-cold PBS and lysed in 0.5 ml IP buffer (25 mM Tris-HCl pH 7.4, 150 mM NaCl, 1% NP-40, 1 mM EDTA, 5% glycerol) containing cOmplete protease inhibitors (Roche). After incubation of cell lysates on ice for 10 min, the cell lysates were centrifuged at 10,000 ***g*** for 10 min at 4°C. The resultant supernatants were incubated with anti-HA antibody for 2 h at 4°C followed by incubation with pre-washed Protein G Sepharose beads (GE Healthcare) for 1 h at 4°C. The beads were washed with IP buffer three times.

### Fractionation

HUVECs cultured in six-well plates were washed with ice-cold PBS and lysed in 0.5 ml IP buffer (25 mM Tris-HCl pH 7.4, 150 mM NaCl, 1% NP-40, 1 mM EDTA, 5% glycerol) containing cOmplete protease inhibitors (Roche). After incubation of cell lysates on ice for 10 min, the cell lysates were centrifuged at 10,000 ***g*** for 10 min at 4°C. The resultant supernatants were then centrifuged at 100,000 ***g*** for 1 h at 4°C.

### Immunofluorescence staining

Cells were fixed with 4% PFA in PBS for 30 min at room temperature and permeabilized with 0.1% Triton X-100 in PBS for 15min at room temperature. For staining of endogenous ANKFY1, cells were fixed with 10% trichloroacetic acid (TCA) in PBS for 15 min at 4°C and permeabilized with 0.05% saponin in PBS for 5 min at room temperature. After blocking with 3% bovine serum albumin (BSA) in PBS for 1 h at room temperature, cells were incubated with primary antibodies and then with secondary antibodies conjugated to fluorophores. To stain nuclei, fixed cells were treated with Hoechst33342 (Dilution 1:2000; Molecular Probes) at room temperature for 1 h.

### Biotinylation assay

For the biotinylation of surface proteins, HUVECs were washed with ice-cold PBS two times followed by incubation with biotin solution (0.1 mg/ml sulfo-NHS-biotin (Thermo Fisher Scientific) in 0.1 M Hepes, 0.15 M NaCl, pH 8.0) for 15 min at 4°C. Cells were quenched with ice-cold serum-containing EBM-2 medium. After wash-out of the remaining biotin solution by ice-cold PBS, cells were subjected to immunoprecipitation as described above with slight modifications. Cell lysates were incubated with streptavidin magnetic beads (Dynabeads M-280, Invitrogen) for 1 h at 4°C. The beads were washed with IP buffer three times, followed by collection of proteins with SDS buffer without 2-mercaptoethanol. Total and biotinylated integrin β1 were detected by western blotting using the TS2/16 antibody.

### Integrin β1 uptake and recycling assay

The internalization and recycling assay of integrin β1 were performed as described previously (Arjonen et al., 2012) with slight modifications. Briefly, integrin β1 on the cell surface of HUVECs was labeled with Alexa488-conjugated TS2/16 antibody in growth medium containing 30 mM Hepes (pH 7.6) on ice for 1 h. Cells were then washed with ice-cold PBS and the medium was replaced with fresh growth medium containing 30 mM Hepes (pH 7.6). For the internalization assay, cells were incubated at 37°C with 5% CO_2_ for the indicated time-point. After internalization, cells were put on ice and the fluorescence on the cell surface was quenched by adding anti-Alexa488 antibody and incubating on ice for 1 h. To monitor the recycling of integrin β1, the cell surface integrin β1 was stained as above. Labeled integrin β1 was allowed to internalize for 1 h at 37°C with 5% CO_2_ followed by quenching of the surface integrin β1 as above. Cells were then re-incubated at 37°C with 5% CO_2_ for the indicated time-point. After re-incubation, the surface fluorescence signal of integrin β1 was quenched again. For imaging, cells were fixed with 4% PFA in PBS for 30 min at room temperature. The fluorescence intensity of Alexa488 minus the background fluorescence intensity was quantified with ImageJ (https://imagej.nih.gov/ij/). Fluorescence intensities were normalized against total surface staining (at 0 min before quenching, for uptake assay) or total internalized staining (for recycling assay).

### Transferrin uptake and recycling assay

The internalization and recycling assay of transferrin were performed as described previously (Lee et al., 2015) with slight modifications. For the uptake assay, HUVECs were serum-starved in EBM-2 for 30 min at 37°C. Cells were then incubated with 50 μg/ml of Alexa488-transferrin (Molecular Probes) in EBM-2 for 5 or 10 min at 37°C. Cells were then chilled on ice and incubated in acid-wash buffer (20 mM sodium-acetate buffer, 1 mM CaCl_2_, 150 mM NaCl, pH 4.8) on ice for 5 min to remove Alexa488-transferrin on the PM. For the recycling assay, HUVECs were incubated in serum-free medium for 30 min at 37°C, followed by incubation in serum-free medium containing 50 μg/ml Alexa488-transferrin for 1 h at 37°C. After washing with ice-cold PBS, cells were incubated in the acid-wash buffer on ice for 5 min to remove surface-bound Alexa488-transferrin. Cells were washed with ice-cold PBS and chased in growth medium containing 400 μg/ml unlabeled human holo-transferrin (Thermo Fisher Scientific) at 37°C with 5% CO_2_. For imaging, cells were fixed with 4% PFA in PBS at room temperature for 30 min. The fluorescence intensity of Alexa488 minus the background fluorescence intensity was quantified with ImageJ.

### Confocal microscopy

Confocal microscopy was performed using an A1R laser confocal microscope (Nikon) with a 60×1.27 Plan-Apochromat water immersion lens or a 10×0.45 Plan-Apochromat lens. Images were analyzed with ImageJ or Fiji (https://fiji.sc) software.

### Adhesion to extracellular matrix

The adhesion assay was performed using a CytoSelect 48-well Cell Adhesion Assay kit (CBA-070, Cell Biolabs, San Diego, USA) according to the manufacturer's instructions. Briefly, HUVECs were collected by treatment with trypsin for 1 min followed by seeding into each well of a 48-well plate (1×10^4^ cells/well). Cells were then incubated in the growth medium for 1 h at 37°C with 5% CO_2_. Adherent cells were measured by staining with a dye followed by measurement at OD560. The data was normalized to the siControl.

### Spreading on basement membrane

HUVECs were collected by treating with trypsin for 1 min followed by seeding on the basement membrane (BD Matrigel™ Basement Membrane Matrix Growth Factor Reduced, BD Biosciences). Cells were then incubated in the growth medium for 1 h at 37°C with 5% CO_2_. Adherent cells were subjected to immunofluorescence staining to label paxillin, viculin, and integrin β1. The cell size was measured with ImageJ.

### Tube formation assay

HUVECs seeded on reconstituted collagen I gel (Nitta Gelatin, Yao, Japan) were subjected to siRNA transfection. At 6 h post-siRNA transfection, cells were packed with collagen I, followed by the addition of 50 ng/ml VEGF-A (R&D Systems) in 0.15% serum-containing EBM-2 medium. Tube formation of HUVECs was observed at 66 h after the addition of VEGF. Cells were fixed with 4% PFA in PBS for 30 min at room temperature and permeabilized with 0.1% Triton X-100 in PBS for 15 min at room temperature. After blocking with 3% BSA in PBS for 30 min at room temperature, cells were incubated with rhodamine-conjugated phalloidin (dilution 1:1000; Molecular Probes) for 1 h at room temperature.

### Statistical analysis

Statistical analysis was carried out by Student's two-tailed *t*-test.

## Supplementary Material

Supplementary information

## References

[BIO029579C1] Arjonen A., Alanko J., Veltel S. and Ivaska J. (2012). Distinct recycling of active and inactive beta1 integrins. *Traffic* 13, 610-625. 10.1111/j.1600-0854.2012.01327.x 22222055 PMC3531618

[BIO029579C2] Arnaoutova I. and Kleinman H. K. (2010). In vitro angiogenesis: endothelial cell tube formation on gelled basement membrane extract. *Nat. Protoc.* 5, 628-635. 10.1038/nprot.2010.6 20224563

[BIO029579C3] Bergers G. and Hanahan D. (2008). Modes of resistance to anti-angiogenic therapy. *Nat. Rev. Cancer* 8, 592-603. 10.1038/nrc2442 18650835 PMC2874834

[BIO029579C4] Carlson T. R., Hu H., Braren R., Kim Y. H. and Wang R. A. (2008). Cell-autonomous requirement for beta1 integrin in endothelial cell adhesion, migration and survival during angiogenesis in mice. *Development* 135, 2193-2202. 10.1242/dev.016378 18480158 PMC2582018

[BIO029579C5] Carmeliet P. and Jain R. K. (2011). Molecular mechanisms and clinical applications of angiogenesis. *Nature* 473, 298-307. 10.1038/nature10144 21593862 PMC4049445

[BIO029579C6] De Franceschi N., Hamidi H., Alanko J., Sahgal P. and Ivaska J. (2015). Integrin traffic - the update. *J. Cell Sci.* 128, 839-852. 10.1242/jcs.161653 25663697 PMC4342575

[BIO029579C7] Eilken H. M. and Adams R. H. (2010). Dynamics of endothelial cell behavior in sprouting angiogenesis. *Curr. Opin. Cell Biol.* 22, 617-625. 10.1016/j.ceb.2010.08.010 20817428

[BIO029579C8] Fischer A., Schumacher N., Maier M., Sendtner M. and Gessler M. (2004). The Notch target genes Hey1 and Hey2 are required for embryonic vascular development. *Genes Dev.* 18, 901-911. 10.1101/gad.291004 15107403 PMC395849

[BIO029579C9] Genschik P., Sumara I. and Lechner E. (2013). The emerging family of CULLIN3-RING ubiquitin ligases (CRL3s): cellular functions and disease implications. *EMBO J.* 32, 2307-2320. 10.1038/emboj.2013.173 23912815 PMC3770339

[BIO029579C10] George E. L., Georges-Labouesse E. N., Patel-King R. S., Rayburn H. and Hynes R. O. (1993). Defects in mesoderm, neural tube and vascular development in mouse embryos lacking fibronectin. *Development* 119, 1079-1091. 8306876 10.1242/dev.119.4.1079

[BIO029579C11] Gschweitl M., Ulbricht A., Barnes C. A., Enchev R. I., Stoffel-Studer I., Meyer-Schaller N., Huotari J., Yamauchi Y., Greber U. F., Helenius A. et al. (2016). A SPOPL/Cullin-3 ubiquitin ligase complex regulates endocytic trafficking by targeting EPS15 at endosomes. *Elife* 5, e13841 10.7554/eLife.13841 27008177 PMC4846373

[BIO029579C12] Hongu T., Funakoshi Y., Fukuhara S., Suzuki T., Sakimoto S., Takakura N., Ema M., Takahashi S., Itoh S., Kato M. et al. (2015). Arf6 regulates tumour angiogenesis and growth through HGF-induced endothelial beta1 integrin recycling. *Nat. Commun.* 6, 7925 10.1038/ncomms8925 26239146

[BIO029579C13] Huotari J., Meyer-Schaller N., Hubner M., Stauffer S., Katheder N., Horvath P., Mancini R., Helenius A. and Peter M. (2012). Cullin-3 regulates late endosome maturation. *Proc. Natl. Acad. Sci. USA* 109, 823-828. 10.1073/pnas.1118744109 22219362 PMC3271891

[BIO029579C14] Hynes R. O. (2002a). Integrins: bidirectional, allosteric signaling machines. *Cell* 110, 673-687. 10.1016/S0092-8674(02)00971-6 12297042

[BIO029579C15] Hynes R. O. (2002b). A reevaluation of integrins as regulators of angiogenesis. *Nat. Med.* 8, 918-921. 10.1038/nm0902-918 12205444

[BIO029579C16] Jin L., Pahuja K. B., Wickliffe K. E., Gorur A., Baumgartel C., Schekman R. and Rape M. (2012). Ubiquitin-dependent regulation of COPII coat size and function. *Nature* 482, 495-500. 10.1038/nature10822 22358839 PMC3292188

[BIO029579C17] Keely P. J., Fong A. M., Zutter M. M. and Santoro S. A. (1995). Alteration of collagen-dependent adhesion, motility, and morphogenesis by the expression of antisense alpha 2 integrin mRNA in mammary cells. *J. Cell Sci.* 108, 595-607. 7769004 10.1242/jcs.108.2.595

[BIO029579C18] Kern A., Eble J., Golbik R. and Kuhn K. (1993). Interaction of type IV collagen with the isolated integrins alpha 1 beta 1 and alpha 2 beta 1. *Eur. J. Biochem.* 215, 151-159. 10.1111/j.1432-1033.1993.tb18017.x 8344274

[BIO029579C19] Khoshnoodi J., Pedchenko V. and Hudson B. G. (2008). Mammalian collagen IV. *Microsc. Res. Tech.* 71, 357-370. 10.1002/jemt.20564 18219669 PMC4788096

[BIO029579C20] Kim S., Bell K., Mousa S. A. and Varner J. A. (2000). Regulation of angiogenesis in vivo by ligation of integrin alpha5beta1 with the central cell-binding domain of fibronectin. *Am. J. Pathol.* 156, 1345-1362. 10.1016/S0002-9440(10)65005-5 10751360 PMC1876892

[BIO029579C21] Lee S., Uchida Y., Wang J., Matsudaira T., Nakagawa T., Kishimoto T., Mukai K., Inaba T., Kobayashi T., Molday R. S. et al. (2015). Transport through recycling endosomes requires EHD1 recruitment by a phosphatidylserine translocase. *EMBO J.* 34, 669-688. 10.15252/embj.201489703 25595798 PMC4365035

[BIO029579C22] Lei L., Liu D., Huang Y., Jovin I., Shai S.-Y., Kyriakides T., Ross R. S. and Giordano F. J. (2008). Endothelial expression of beta1 integrin is required for embryonic vascular patterning and postnatal vascular remodeling. *Mol. Cell. Biol.* 28, 794-802. 10.1128/MCB.00443-07 17984225 PMC2223431

[BIO029579C23] Lupo G., Caporarello N., Olivieri M., Cristaldi M., Motta C., Bramanti V., Avola R., Salmeri M., Nicoletti F. and Anfuso C. D. (2016). Anti-angiogenic therapy in cancer: downsides and new pivots for precision medicine. *Front Pharmacol* 7, 519 10.3389/fphar.2016.00519 28111549 PMC5216034

[BIO029579C24] Muller P. A. J., Caswell P. T., Doyle B., Iwanicki M. P., Tan E. H., Karim S., Lukashchuk N., Gillespie D. A., Ludwig R. L., Gosselin P. et al. (2009). Mutant p53 drives invasion by promoting integrin recycling. *Cell* 139, 1327-1341. 10.1016/j.cell.2009.11.026 20064378

[BIO029579C25] Nehru V., Voytyuk O., Lennartsson J. and Aspenström P. (2013). RhoD binds the Rab5 effector Rabankyrin-5 and has a role in trafficking of the platelet-derived growth factor receptor. *Traffic* 14, 1242-1254. 10.1111/tra.12121 24102721

[BIO029579C26] Ohnuki H., Inoue H., Takemori N., Nakayama H., Sakaue T., Fukuda S., Miwa D., Nishiwaki E., Hatano M., Tokuhisa T. et al. (2012). BAZF, a novel component of cullin3-based E3 ligase complex, mediates VEGFR and Notch cross-signaling in angiogenesis. *Blood* 119, 2688-2698. 10.1182/blood-2011-03-345306 22279058

[BIO029579C27] Petroski M. D. and Deshaies R. J. (2005). Function and regulation of cullin-RING ubiquitin ligases. *Nat. Rev. Mol. Cell Biol.* 6, 9-20. 10.1038/nrm1547 15688063

[BIO029579C28] Potente M., Gerhardt H. and Carmeliet P. (2011). Basic and therapeutic aspects of angiogenesis. *Cell* 146, 873-887. 10.1016/j.cell.2011.08.039 21925313

[BIO029579C29] Ramasamy S. K., Kusumbe A. P. and Adams R. H. (2015). Regulation of tissue morphogenesis by endothelial cell-derived signals. *Trends Cell Biol.* 25, 148-157. 10.1016/j.tcb.2014.11.007 25529933 PMC4943524

[BIO029579C30] Sakaue T., Fujisaki A., Nakayama H., Maekawa M., Hiyoshi H., Kubota E., Joh T., Izutani H. and Higashiyama S. (2017a). Neddylated Cullin 3 is required for vascular endothelial-cadherin-mediated endothelial barrier function. *Cancer Sci.* 108, 208-215. 10.1111/cas.13133 27987332 PMC5329144

[BIO029579C31] Sakaue T., Maekawa M., Nakayama H. and Higashiyama S (2017b). Prospect of divergent roles for the CUL3 system in vascular endothelial cell function and angiogenesis. *J. Biochem.* 162, 237-245. 10.1093/jb/mvx051 28981750

[BIO029579C32] Schnatwinkel C., Christoforidis S., Lindsay M. R., Uttenweiler-Joseph S., Wilm M., Parton R. G. and Zerial M. (2004). The Rab5 effector Rabankyrin-5 regulates and coordinates different endocytic mechanisms. *PLoS Biol.* 2, E261 10.1371/journal.pbio.0020261 15328530 PMC514490

[BIO029579C33] Senger D. R., Claffey K. P., Benes J. E., Perruzzi C. A., Sergiou A. P. and Detmar M. (1997). Angiogenesis promoted by vascular endothelial growth factor: regulation through alpha1beta1 and alpha2beta1 integrins. *Proc. Natl. Acad. Sci. USA* 94, 13612-13617. 10.1073/pnas.94.25.13612 9391074 PMC28354

[BIO029579C34] Soucy T. A., Smith P. G., Milhollen M. A., Berger A. J., Gavin J. M., Adhikari S., Brownell J. E., Burke K. E., Cardin D. P., Critchley S. et al. (2009). An inhibitor of NEDD8-activating enzyme as a new approach to treat cancer. *Nature* 458, 732-736. 10.1038/nature07884 19360080

[BIO029579C35] Stogios P. J., Downs G. S., Jauhal J. J., Nandra S. K. and Prive G. G. (2005). Sequence and structural analysis of BTB domain proteins. *Genome Biol.* 6, R82 10.1186/gb-2005-6-10-r82 16207353 PMC1257465

[BIO029579C36] Sun Z., Guo S. S. and Fässler R. (2016). Integrin-mediated mechanotransduction. *J. Cell Biol.* 215, 445-456. 10.1083/jcb.201609037 27872252 PMC5119943

[BIO029579C37] Tanjore H., Zeisberg E. M., Gerami-Naini B. and Kalluri R. (2008). Beta1 integrin expression on endothelial cells is required for angiogenesis but not for vasculogenesis. *Dev. Dyn.* 237, 75-82. 10.1002/dvdy.21385 18058911

[BIO029579C38] Tiwari A., Jung J.-J., Inamdar S. M., Brown C. O., Goel A. and Choudhury A. (2011). Endothelial cell migration on fibronectin is regulated by syntaxin 6-mediated alpha5beta1 integrin recycling. *J. Biol. Chem.* 286, 36749-36761. 10.1074/jbc.M111.260828 21880737 PMC3196105

[BIO029579C39] Wimuttisuk W. and Singer J. D. (2007). The Cullin3 ubiquitin ligase functions as a Nedd8-bound heterodimer. *Mol. Biol. Cell* 18, 899-909. 10.1091/mbc.E06-06-0542 17192413 PMC1805106

[BIO029579C40] Wu J.-T., Lin H.-C., Hu Y.-C. and Chien C.-T. (2005). Neddylation and deneddylation regulate Cul1 and Cul3 protein accumulation. *Nat. Cell Biol.* 7, 1014-1020. 10.1038/ncb1301 16127432

[BIO029579C41] Yamamoto H., Ehling M., Kato K., Kanai K., van Lessen M., Frye M., Zeuschner D., Nakayama M., Vestweber D. and Adams R. H. (2015). Integrin beta1 controls VE-cadherin localization and blood vessel stability. *Nat. Commun.* 6, 6429 10.1038/ncomms7429 25752958

[BIO029579C42] Yang J. T., Rayburn H. and Hynes R. O. (1993). Embryonic mesodermal defects in alpha 5 integrin-deficient mice. *Development* 119, 1093-1105. 7508365 10.1242/dev.119.4.1093

[BIO029579C43] Yao W. T., Wu J. F., Yu G. Y., Wang R., Wang K., Li L. H., Chen P., Jiang Y. N., Cheng H., Lee H. W. et al. (2014). Suppression of tumor angiogenesis by targeting the protein neddylation pathway. *Cell Death Dis.* 5, e1059 10.1038/cddis.2014.21 24525735 PMC3944239

[BIO029579C44] Yuan W.-C., Lee Y.-R., Lin S.-Y., Chang L.-Y., Tan Y. P., Hung C.-C., Kuo J.-C., Liu C.-H., Lin M.-Y., Xu M. et al. (2014). K33-linked polyubiquitination of coronin 7 by Cul3-KLHL20 ubiquitin E3 ligase regulates protein trafficking. *Mol. Cell* 54, 586-600. 10.1016/j.molcel.2014.03.035 24768539

[BIO029579C45] Yurchenco P. D. (2011). Basement membranes: cell scaffoldings and signaling platforms. *Cold Spring Harb. Perspect. Biol.* 3, a004911 10.1101/cshperspect.a004911 21421915 PMC3039528

[BIO029579C46] Zhang J., Reiling C., Reinecke J. B., Prislan I., Marky L. A., Sorgen P. L., Naslavsky N. and Caplan S. (2012). Rabankyrin-5 interacts with EHD1 and Vps26 to regulate endocytic trafficking and retromer function. *Traffic* 13, 745-757. 10.1111/j.1600-0854.2012.01334.x 22284051 PMC3613124

[BIO029579C47] Zhao Y. and Sun Y. (2013). Cullin-RING ligases as attractive anti-cancer targets. *Curr. Pharm. Des.* 19, 3215-3225. 10.2174/13816128113199990300 23151137 PMC4034125

[BIO029579C48] Zovein A. C., Luque A., Turlo K. A., Hofmann J. J., Yee K. M., Becker M. S., Fassler R., Mellman I., Lane T. F. and Iruela-Arispe M. L. (2010). Beta1 integrin establishes endothelial cell polarity and arteriolar lumen formation via a Par3-dependent mechanism. *Dev. Cell* 18, 39-51. 10.1016/j.devcel.2009.12.006 20152176 PMC3178410

