# Supplementary material for: Cullin-3 and its adaptor protein ANKFY1 determine the surface level of integrin β1 in endothelial cells
Source: Biol Open. 2017 Oct 16;6(11):1707–19. doi: 10.1242/bio.029579 (PMC5703617; doi:10.1242/bio.029579)
Supplement: Supplementary information [file biolopen-6-029579-s1.pdf]

Figure S1

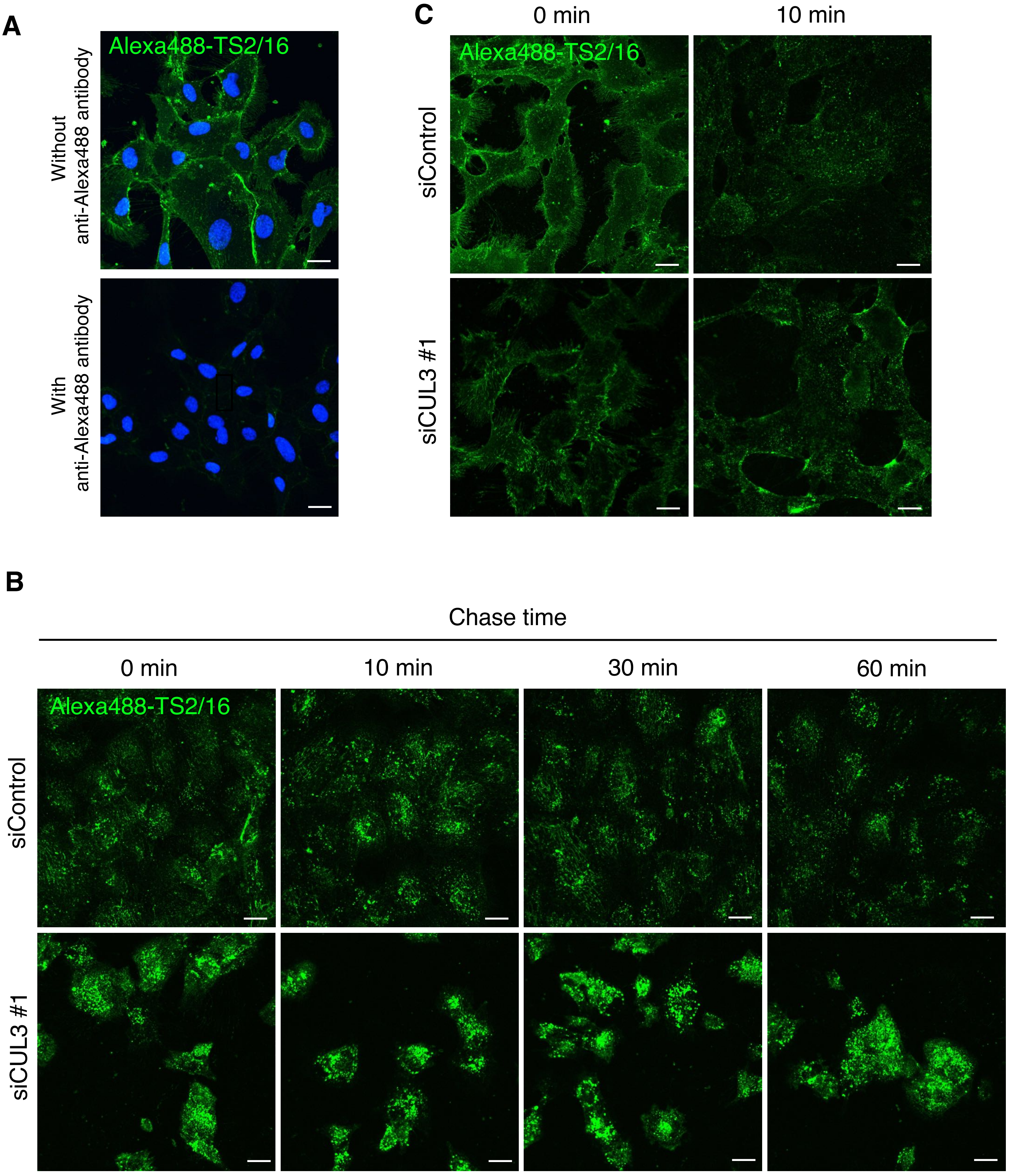

Fig. S1. Images of integrin β1 trafficking assay. (A) HUVECs were treated with Alexa488-TS2/16 for 1 h on ice to label surface integrin β1 (left) and subsequently cells were incubated with anti-Alexa488 antibody on ice for 1 h to quench the fluorescence signal of Alexa488-TS2/16 on the cell surface (right). Bars; 20 μm. (B) Representative images of the recycling assay of integrin β1 in Fig. 2E. Bars; 20 μm. (C) Representative images of the internalization assay of integrin β1 in Fig. 2F. Bars; 20 μm.

Biology Open • Supplementary information

Figure S2

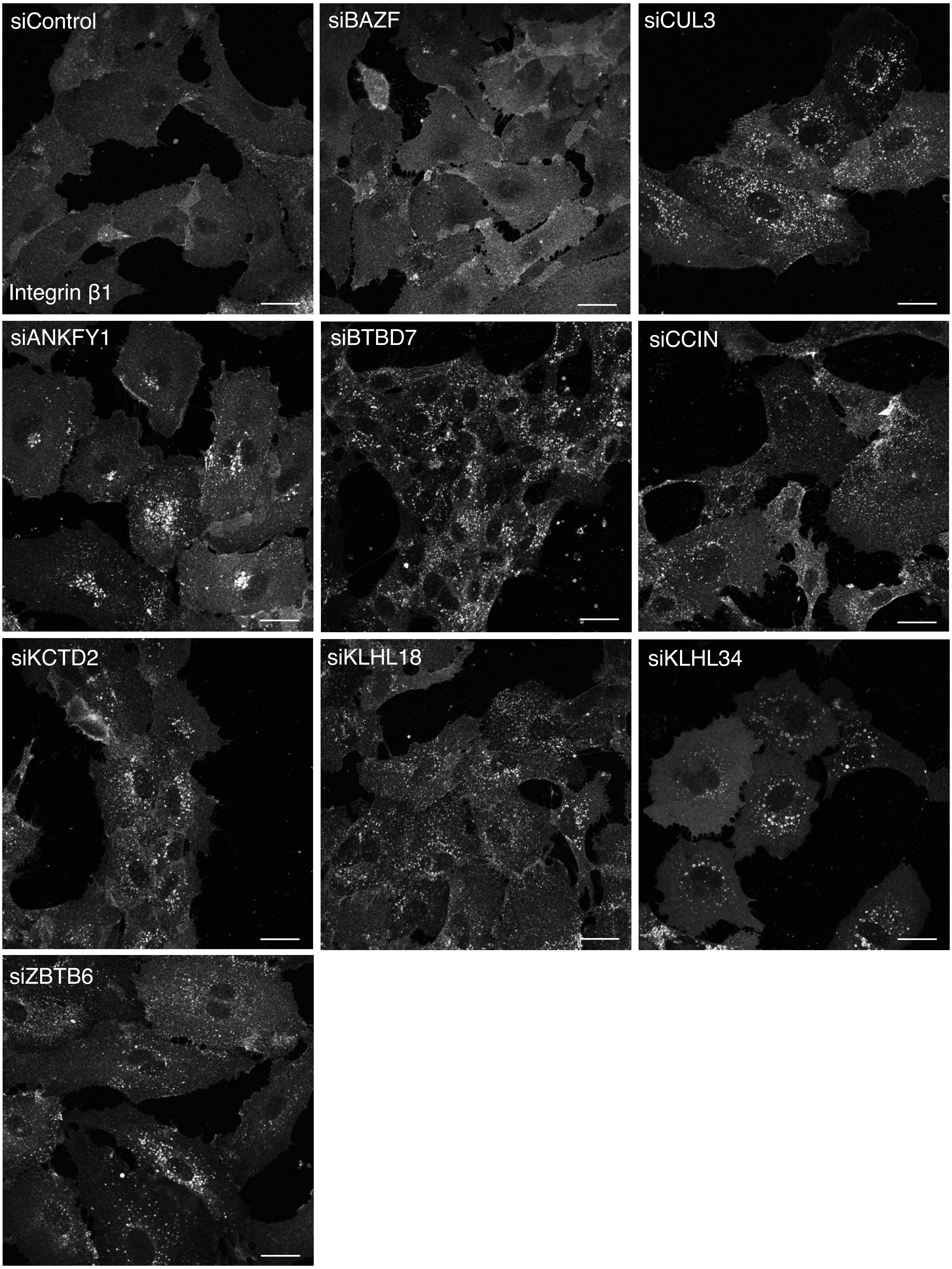

Fig. S2. Representative images of BTBP siRNA screening. Confocal images of intracellular integrin β1 in HUVECs treated with siRNA targeting BAZF, CUL3, ANKFY1, BTBD7, CCIN, KCTD2, KLHL18, KLHL34, and ZBTBT6. The sequences of siRNAs are shown in Table S1. Bars; 20 µm.

Biology Open • Supplementary information

Figure S3

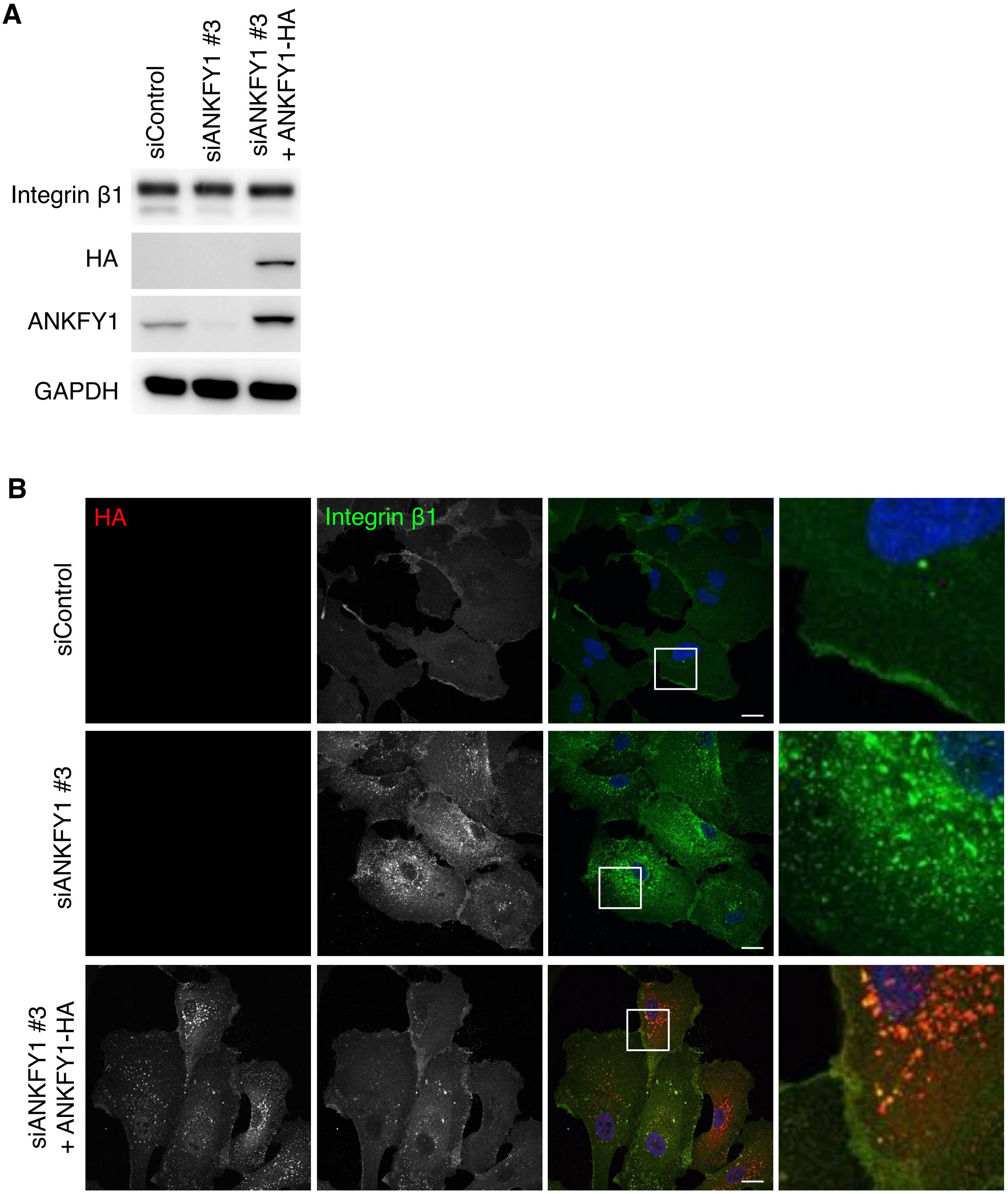

Fig. S3. Rescue experiments for ANKFY1 knockdown. (A) Western blots of cell lysates of HUVECs infected with siRNA resistant-ANKFY1-HA-carrying lentivirus. (B) Confocal images of intracellular integrin β1 in HUVECs infected with siRNA-resistant-ANKFY1-HA-carrying lentivirus. Integrin β1 and ANKFY1-HA were labelled with P5D2 and anti-HA antibody, respectively. Magnifications of the squared areas are shown on the right. Bars; 20 µm.

Biology Open • Supplementary information

Figure S4

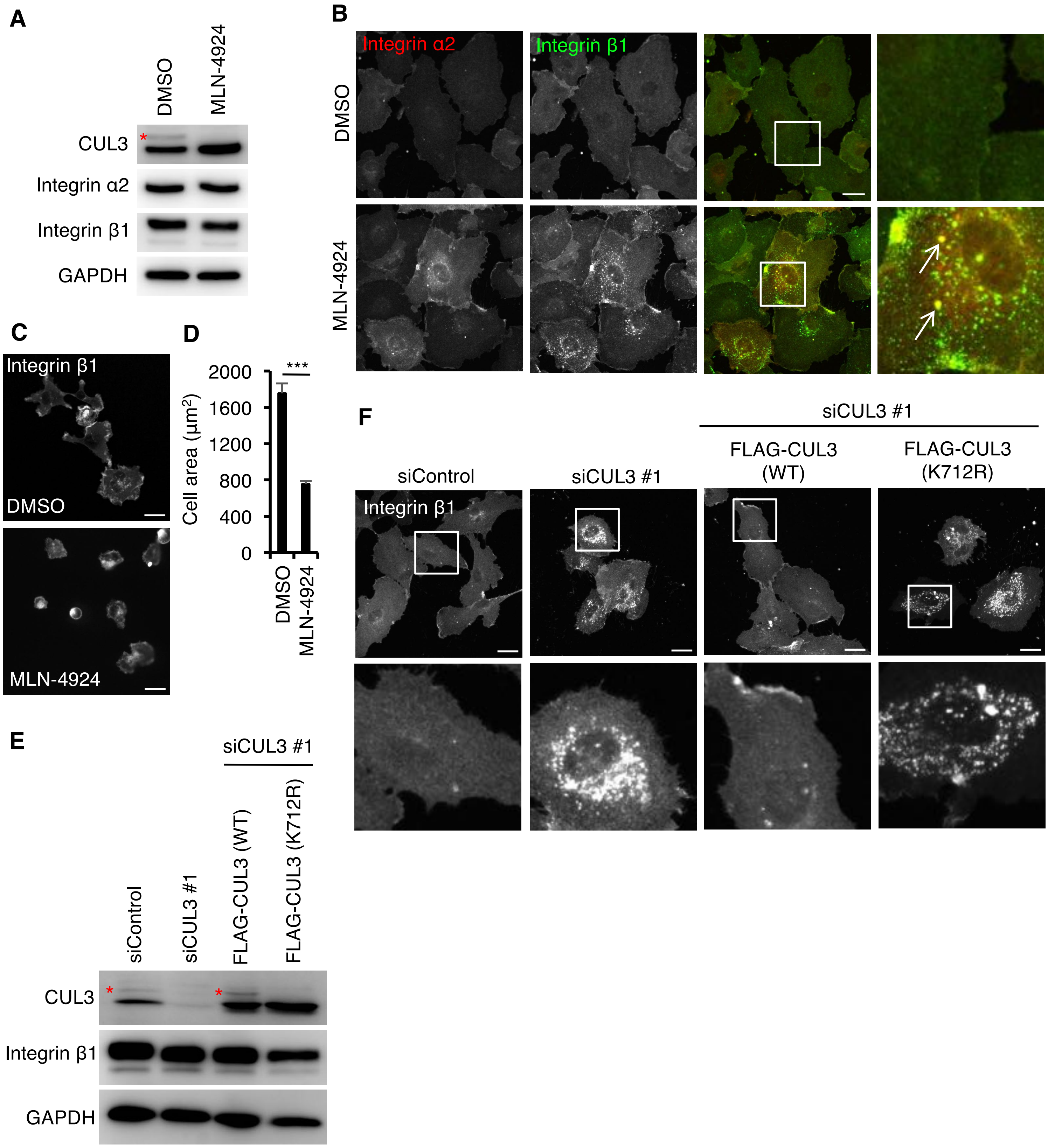

Biology Open • Supplementary information

Fig. S4. Neddylation of CUL3 determines the cellular distribution of integrin β1 and spreading on the basement membrane. (A) Western blots of cell lysates of HUVECs treated with 1 µM MLN-4924 for 20 h. The asterisk indicates neddylated CUL3. (B) Confocal images of intracellular integrin β1 and α2. HUVECs treated with 1 µM MLN-4924 for 20 h were fixed, permeabilized, and stained for integrin β1 and α2 by P5D2 and anti-integrin α2 antibody, respectively. Magnifications of the squared areas are shown on the right. Representative colocalized integrin β1 and α2 are indicated by arrows. Bars; 20 µm. (C) Confocal images of HUVECs during spreading on the basement membrane. HUVECs with 1 µM MLN-4924 for 20 h were seeded on the basement membrane, incubated for 1 h, fixed, and subjected to immunofluorescence staining. Integrin β1 was labelled with P5D2. Bars; 20 µm. (D) Cell size of (C) are shown. More than seventy cells from three independent experiments were analyzed. Data show the mean ± sem. ***, $p<0.001$. (E) Western blots of cell lysates of HUVECs infected with siRNA-resistant-FLAG-CUL3 (WT or K712R)-carrying lentivirus. The asterisks indicate neddylated CUL3. (F) Confocal images of intracellular integrin β1 in HUVECs infected with siRNA resistant-FLAG-CUL3 (WT or K712R)-carrying lentivirus. Integrin β1 was labelled with P5D2 antibody. Magnifications of squared areas are shown in the lower panels. WT; wild-type, K712R; non-neddylated mutant. Bars; 20 µm.

Biology Open • Supplementary information

Figure S5

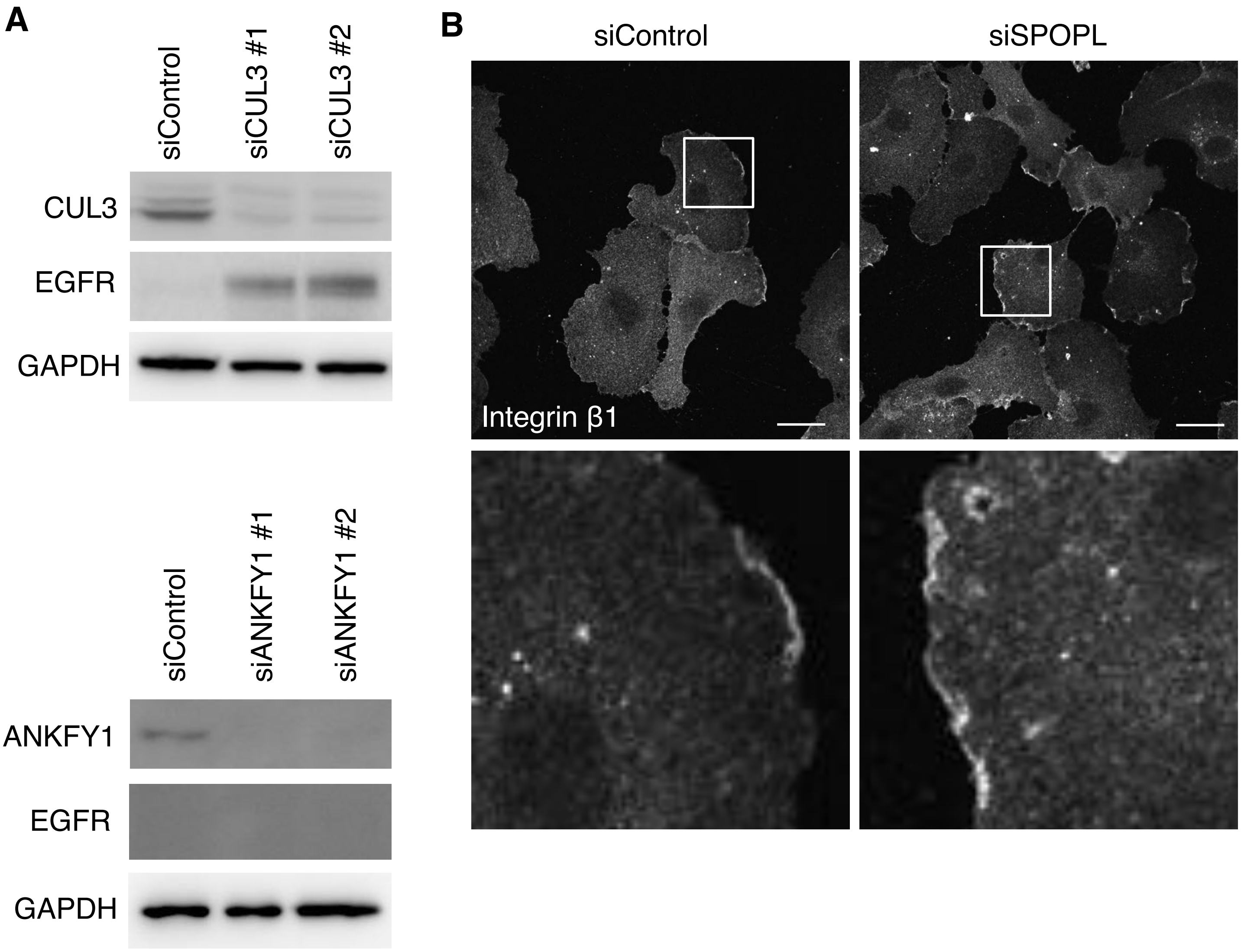

Fig. S5. Effects of CUL3, ANKFY1, or SPOPL knockdown on EGFR expression or cellular distribution of integrin β1 in HUVECs. (A) Western blots of cell lysates of HUVECs at 72 h post-transfection of siRNAs. (B) Confocal images of intracellular integrin β1 in HUVECs treated with siRNA targeting SPOPL. The sequence of SPOPL siRNA is shown in Table S1. Bars; 20 μm.

Biology Open • Supplementary information

Figure S6

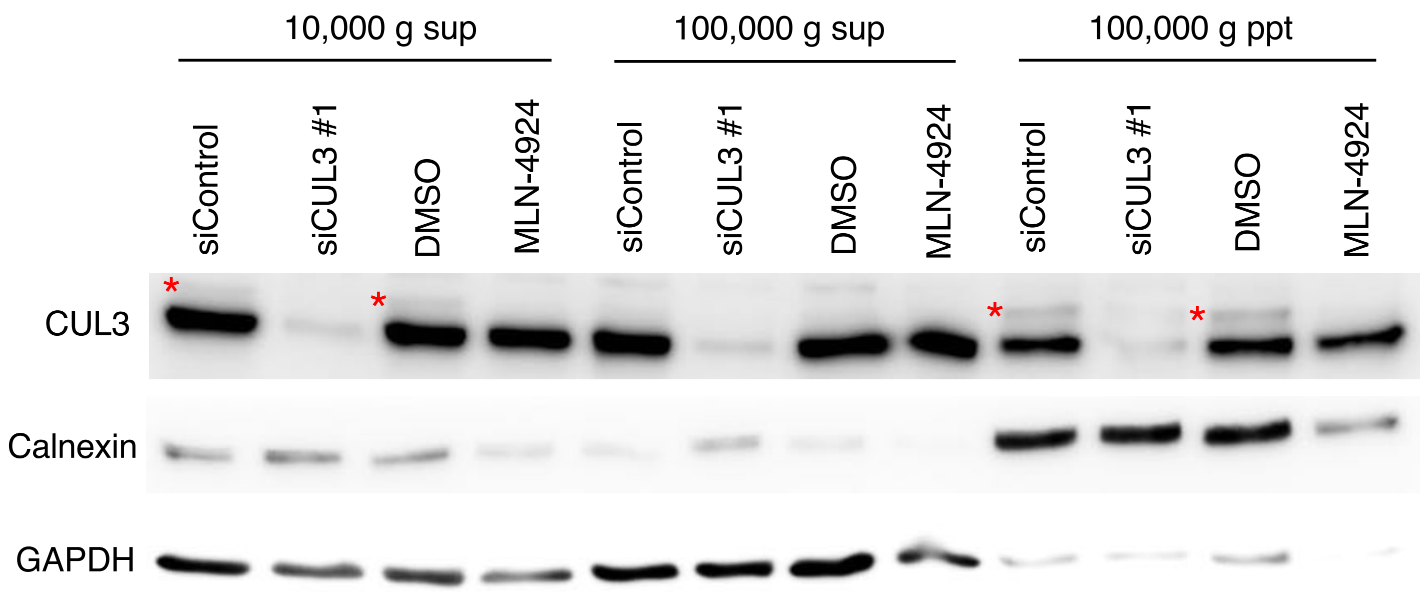

Fig. S6. Fractionation of CUL3 in cytosol and membrane fraction from cell lysate of HUVECs. Western blots of cell lysates (10,000 g sup), cytosol fraction (100,000 g sup), and membrane fraction (100,000 g ppt) of HUVECs are shown. HUVECs were treated with control siRNA and CUL3 siRNA #1 for 72 h, DMSO, and 1 μM MLN-4924 for 20 h before fractionation. Anti-calnexin and anti-GAPDH antibody were used as membrane and cytosol markers, respectively. The asterisks indicate neddylated CUL3.

Table S1. List of BTBP genes and sequences of siRNAs used for the siRNA screening.

Click here to Download Table S1

Biology Open • Supplementary information
